# Biocompatible Polymers Combined with Cyclodextrins: Fascinating Materials for Drug Delivery Applications

**DOI:** 10.3390/molecules25153404

**Published:** 2020-07-28

**Authors:** Bartłomiej Kost, Marek Brzeziński, Marta Socka, Małgorzata Baśko, Tadeusz Biela

**Affiliations:** Centre of Molecular and Macromolecular Studies, Polish Academy of Sciences, Sienkiewicza 112, 90-363 Lodz, Poland; msocka@cbmm.lodz.pl (M.S.); baskomeg@cbmm.lodz.pl (M.B.); tadek@cbmm.lodz.pl (T.B.)

**Keywords:** polylactide, cyclodextrin, drug delivery systems, controlled release

## Abstract

Cyclodextrins (CD) are a group of cyclic oligosaccharides with a cavity/specific structure that enables to form inclusion complexes (IC) with a variety of molecules through non-covalent host-guest interactions. By an elegant combination of CD with biocompatible, synthetic and natural polymers, different types of universal drug delivery systems with dynamic/reversible properties have been generated. This review presents the design of nano- and micro-carriers, hydrogels, and fibres based on the polymer/CD supramolecular systems highlighting their possible biomedical applications. Application of the most prominent hydrophobic aliphatic polyesters that exhibit biodegradability, represented by polylactide and polycaprolactone, is described first. Subsequently, particular attention is focused on materials obtained from hydrophilic polyethylene oxide. Moreover, examples are also presented for grafting of CD on polysaccharides. In summary, we show the application of host-guest interactions in multi-component functional biomaterials for controlled drug delivery.

## 1. Introduction

**Figure 1:**
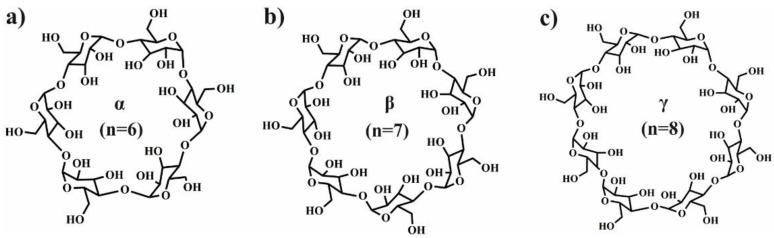
The structure of (**a**) α-CD, (**b**) β-CD, (**c**) γ-CD, n-number of glucopiranose units.

Supramolecular chemistry aims to construct highly complex, functional systems through a variety of noncovalent interactions (e.g., multiple hydrogen bonding, metal coordination, host–guest interactions, and aromatic stacking) [1]. The discovery of host-guest recognition that mimics biological structure formation and their functions [2,3], led to the development of numerous supramolecular systems. For instance, the host-guest interactions provide a possibility to integrate two or more chemical moieties in a supramolecular system [4,5]. In this review, among the many investigated host molecules, we will focus on the cyclodextrins (CDs), which belongs to the family of cyclic oligomers composed of α-(1→4)-linked glucose units in 4C_10_ chair conformation [4]. Depending on the number of glucose units, the ones containing 6, 7, and 8 are named as α-, β-, and γ-CD as shown in Figure 1 [6], respectively. The structure of CDs is described as a truncated cone with a hydrophilic outer surface and hydrophobic core that allows for the encapsulation of a variety of bioactive guest molecules. The formation of inclusion complex is based on non-covalent bonds such as hydrogen or van der Waals bonds. The main driving force of IC formation is the release of the water molecules from the hydrophilic cavity. The water is replaced by more hydrophilic compounds present in the solution and the apolar-apolar interaction is formed. The formation of this type of interaction causes decreasing of CDs ring strain and resulting in more favourable and lower energy state. The list of potential guest which could be encapsulated inside the CDs is quite varied and included low molecular weight molecules such aldehydes, organic acid, ketones, fatty acids, amines and linear or branched polymers. Due to the size of the inner cavity of CDs, different CDs can complex different molecules, for instance: α-CD can typically complex low molecular weight molecules or aliphatic polymer chains, β-CD will complex aromatics or heterocycles compounds, and γ-CD can entrap the largest molecules such as macrocycles or steroids. Moreover, the hydroxyl functionalities present at CDs surface can be used as a initiating groups in ring-opening polymerization (ROP) of cyclic esters, leading to functionalized polyesters with the core able to form IC [7]. In addition, the grafting to methods (e.g., click reaction [8], coupling [9]) can be used to combine polyesters or polyethers with CDs. Moreover, grafting from and grafting to methods are also used to combine the CDs with saccharides and polysaccharides [10]. All these methods lead to the formation of well-defined and relatively uniform polymers with the ability to form host-guest interactions. The growing interest in the application of macrocyclic molecules in combination with biodegradable polymers, especially with CDs, is related with their biocompatibility and reversibility of host-guest interactions which leads to the stimuli-responsive supramolecular systems [11,12]. This feature is closely related to the kinetic and thermodynamic properties of host−guest complexes and it is determined by the sizes of host and guest, as well as the environmental conditions such as pH and temperature [13]. Therefore, a large amount of efforts was focused on the utilization of this phenomenon in the control drug release of CD-based supramolecular systems. This review focuses on the highly organized drug delivery systems which combines biocompatible synthetic polymers/biopolymers with CDs and discusses the fabrication and biomedical applications of supramolecular systems based on host−guest interactions (with the exclusion of non-degradable polymers and cellulose since the excellent review about cellulose/CDs is already existing in the literature [14]. Therefore, we group the research work into four sections which describe the drug delivery systems containing CD combined with polylactide, poly(ε-caprolactone), poly(ethylene glycol), and polysaccharides. The specific biomedical applications of the host−guest systems based on these polymers are discussed with the focus on several leading directions, that is, drug delivery, gene delivery, antibacterial activity, bone regeneration, photodynamic therapy, tumour targeting, wound healing, and removal of micropollutants. The design and functions of nano- and micro-carriers, hydrogels, and fibres based on the CD-based supramolecular systems have been highlighted.

## 2. Polylactide Systems Based on Cyclodextrin for Controlled Drug Delivery

**Figure 2:**
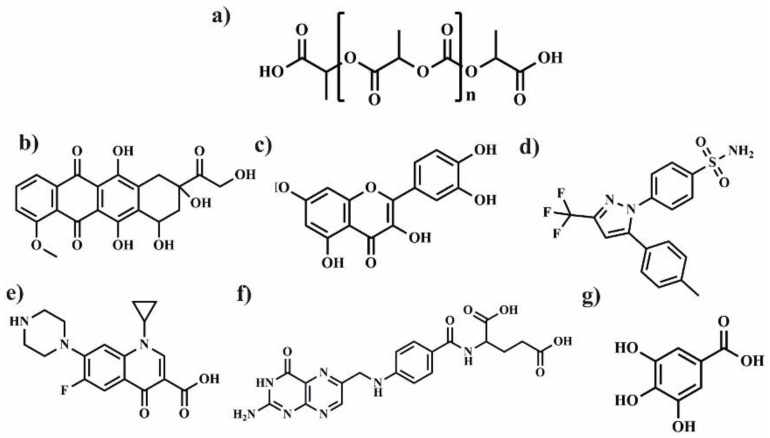
The chemical structure of (**a**) the carrier polymer-PLA, and chemical structure of (**b**) DOX, (**c**) quercetin, (**d**) celecoxib, (**e**) ciprofloxacine, (**f**) folic acid, (**g**) gallic acid.

Polylactide (PLA) (Figure 2) is biodegradable aliphatic polyester that can be produced from naturally occurring renewable resources, such as corn or sugar beets. Due to its biocompatibility and ability to degradation to non-toxic products, PLA can be excellent platform for the preparation of various polymeric drug delivery systems [15]. However, some applications of PLA are limited because of its low solubility in water, long degradation time, and weak encapsulation of polar drug. To overcome these drawbacks, LA is copolymerized with chosen kind of monomers [16] or PLA is connected with polyethylene glycol (PEG) [17]. Despite these disadvantages, PLA and its copolymers are frequently used for biomedical applications for nanoparticles (NPs), microparticles (MPs), fibres or hydrogels preparation. In this part of review, the application of polymeric drug delivery systems such as NPs, MPs, hydrogels and fibres using a combination of cyclodextrin, polylactide, and its copolymers is described and summarized in Table 1.

**Figure 3:**
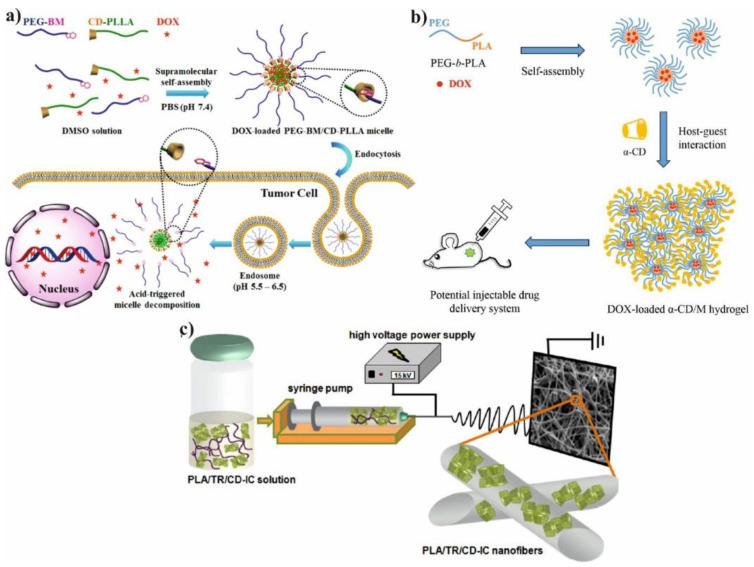
Schematic illustration of formation (**a**) nanoparticles, (**b**) hydrogels and, (**c**) fibres based on PLA and its copolymers for drug delivery applications. (Copyrights 2019 ACS (1A), Copyrights 2018 Elsevier (1B), Copyrights 2013 ACS (1C)).

Supramolecular hydrogels are extensively used as a promising tools for drug delivery systems due to their ability to control release of drug, absorb large amount of water and low toxicity. Polyglycolide (PGA) or polyethylene glycol (PEG) are hydrophilic polymers widely used for preparation PLA-based hydrogels [18]. Also triblock copolymer of poly(lactide-*co*-glycolide-*co*-ethylene glycol) (PLGA-PEG-PLGA) was used to prepare supramolecular hydrogel due to its good solubility in water. However, the high *M_n_* of PEG and low LA/GA ratio is crucial to maintain hydrophilic nature of PLGA-PEG-PLGA copolymer. After mixing of a solution of triblock copolymer with solution of α-CD in water, the gelation process occurs as a result of IC formation between PLGA chain and cyclodextrin cavity. The gelation time can be reduced by increasing the α-CD concentration. In addition, the increasing of hydrophilic-lipophilic balance causes shorter gelation time and faster model drug e.g., vitamin B_12_ (B_12_) releases. Moreover, the alteration of the ratio of PLGA-PEG-PLGA to α-CD leads to controlled release of B_12_ from supramolecular hydrogel [19]. Therefore, the hydrogels could be also obtained by treating the solution of diblock copolymer PLA-*b*-PEG with an α-CD solution (Figure 3b). The core-shell structure of hydrogel was achieved due to the amphiphilic nature of PLA-*b*-PEG copolymer and their micellar aggregation. The aggregation process is driven by the spontaneous self-assembly of the polymer in water. Therefore, as a result of this process hydrophobic part of copolymer (PLA) creates inner core while hydrophilic part (PEG) forms outer corona. However, the two types of physical interactions are required for gelation: association of PLA-PEG micelles and formation of IC microcrystals. The formation of micellar hydrogels depend on several factors such as α-CD concentration, polymer concentration, and temperature of the process. The selection of proper parameters is essential to prepare high-quality hydrogels. The hydrogels were also loaded with doxorubicin (DOX) and its release profiles were evaluated in PBS at 37 °C with varying type of hydrogels formulation. The release rate of DOX from hydrogels decreases as the concentration of α-CD increases because of enhancement in hydrogel strength. It is worth noting that blank micellar hydrogel is non-toxic against HeLa cells, and after DOX encapsulation the hydrogel efficiently delivers cargo to the desired target. It was confirmed by the uptake analysis, which showed that DOX can be located in nucleus and cytoplasm through the carrier–mediated endocytosis pathway [20].

**Table 1:** The summary of described hydrogels, microparticles and fibres for drug delivery based on combination of PLA and its copolymers with different CDs.

Type of Drug Delivery System	Platform	Type of CD	Drug	Release Medium	In Vitro Studies	Ref.
Hydrogel	PLGA-PEG	α-CD	Vitamin B12	PBS	nd	[19]
Hydrogel	PLA-PEG	α-CD	DOX	PBS	HeLa cells	[20]
Fibre	PLA	γ-CD	Gallic acid	10% or 95% EtOH	nd	[21]
Fiber	PLA	Mβ-CD	Quercetin	PBS	nd	[22]
Fibre	PLA	polyCD	Ciprofloxaxin	PBS	NIH3T3 cells	[23]
Microparticle	PLGA	γ-CD	Dexamethasone	PBS	nd	[24]
Microparticle	PLGA	DMβ-CD	Celecoxib	PBS	Human chondrocytes	[25]
Microparticle	PLGA	HPβ-CD	Prostaglandin	PBS	Calu-3 cells	[26]
Nanoparicle	PLGA	HPβ-CD	Triamcinolone acetonide	PBS	Rabbit eyes	[27]
Nanoparicle	PLA-PEG	β-CD	Folic acid	nd	HEK293T cells	[28]
Nanoparticle	PLA/PEG	β-CD	DOX	pH 6.0, pH 5.5, pH 7.4	HepG2 cells	[29]

**Figure 4:**
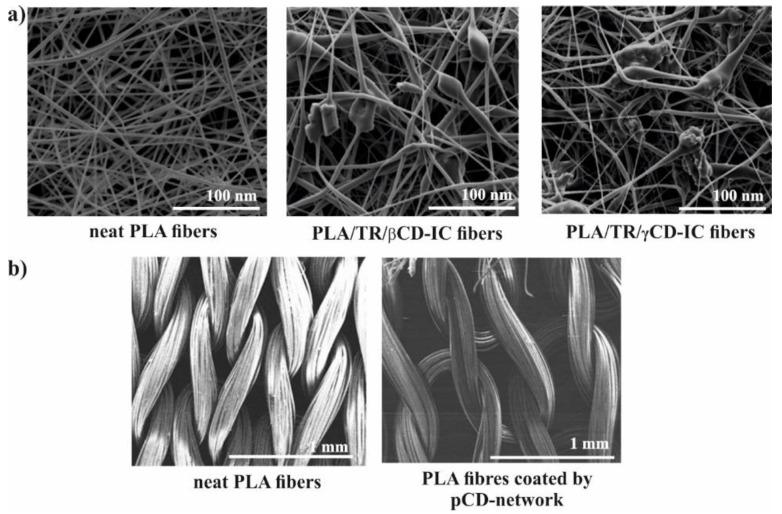
The SEM imaging of PLA fibres with (**a**) TR inclusion complex and (**b**) coated by pCD-citrate network for antibacterial drugs delivery. (Copyrights 2013 ACS (**a**), Copyrights 2017 Acta Materialia (**b**)).

The well-known electrospinning process can be useful method for production of microfibers (MF) or nanofibers (NF) for biomedical application as shown in Figure 3c. Uyar and co-workers used linear poly(lactide) (PLA) modified with CD to prepare nanofibers loaded with two different antioxidant agents: gallic acid (GA), tocopherol (TC) or an antibiotic: triclosan (TR). The electrospinning allows for the preparation PLA nanofibers with incorporated IC between GA or TC and γ-CD (Figure 4a). The molecular modelling was employed to confirm the ability of GA to IC formation. The penetration of cyclodextrin cavity by GA leads to the formation of two energetically stable structures with relatively low energy. The release studies showed that dosage control of GA and TC strongly depends on release media, nanofiber diameter and solubility of GA. Additionally, due to the antioxidant properties of GA or TC, the radical scavenging assay was performed. However, the GA incorporated in γ-CD showed slightly lower activity than free GA due to the specific orientations of GA hydroxyl group in γ-CD cavity. Therefore, despite faster release of tocopherol (α-TC) from PLA/α-TC/γ-CD-IC-NF, in comparison to α-TC-loaded PLA nanofibres, there was no significant difference in antioxidant activity and lipid oxidation inhibition between both systems. The TR- loaded fibres showed the broad spectrum of activity against gram-positive and gram-negative bacteria. Due to the well-known antibacterial properties of TR, the PLA-TR nanofibres cause the inhibition in the growth of both *E. coli* and *S. aureus* bacteria strains. The observed inhibition zone around the fibres is wider in the case of polylactide NF with IC (PLA-CD/TR) than with free TR what can be related to better solubility of CD/TR systems in agar media. Most importantly, the better inhibitions level against bacteria was observed for NF with the β-CD IC. The difference between β-CD and γ-CD systems may be caused by the partly uncomplexed TR in the case of β-CD, and uncomplexed TR can affect the bacteria at the initial stage before the complexed drug release from NF [21,30,31].

Microfibers (MF) with β-CD were used as an excellent platform for quercetin (Q) delivery. Kost et al. have prepared PLAs with ability to formation of IC due to presence of β-CD in the polymer core. The presence of β-CD covalently built into polymer structure allow for creation of IC between β-CD-PLA and Q. Electrospinning of such modified polylactides leads to formation of various types of Q-loaded supramolecular MF with antibacterial activity. Importantly, MF forms Q protective scaffold against the destructive effects of light and oxygen. In contrast to TR-loaded microfibers, in this case the release of Q to the phosphate buffer saline (PBS) or agar plate was not observed. Moreover, the inhibition zone appears only where the materials come into direct contact with the agar medium. The delayed release of Q is associated with a strong entrapment of Q in the fibres or in the cyclodextrin cavity, poor solubility of Q in water and the hydrophobic nature of PLA. Despite the fact that all nonwovens with Q loading were yellow, after rubbing against the skin or white paper they did not leave yellow marks, which is important from a practical point of view when we intend to use them as dressing material [22]. To prepare PLA fabrics covered by a β-CD network with prolonged antibacterial activity, the pad/dry/cure technique was successfully employed (Figure 4b). The PLA was impregnated in a water solution containing CDs, citric acid and catalyst. After extraction of unreacted components (130 °C), the PLA textiles covered with β-CD network were prepared (PLA/β-CD). The ciprofloxacin-loaded PLA/β-CD textiles after exposition on two strains (*E. coli, S. aureus*) sufficiently reduced the number of bacteria. Moreover, the increasing of β-CD ratio in PLA/β-CD textiles from 8% to 33%, provided prolonged antibacterial activity up to 24 h and 120 h for *S. aureus* and *E. coli*, respectively. Additionally, PLA/β-CD textiles were estimated as a non-toxic against mouse fibroblast after 3 h. After 6 days, the viability of cells was reduced to 65% and decreased with increasing of cyclodextrin concertation. The possible degradation of cyclodextrin network by hydrolysis of esters bond of PLA produces free carboxylic acid and thus faster cell proliferation [23]. Due to their biocompatibility and biodegradability, poly(lactide-*co*-caprolactone) (PLA-*co*-PCL) was chosen to produce nanofibres scaffold for the inhibition of growth MCF-7 cells. The different combinations of MgO nanoparticles (MgO NPs), curcumin (Cur) and aloe vera (AV) allow for producing various types of drug delivery nanofibres scaffold via electrospinning. The MgO NPs can be mixed with β-CD and Cur to form multitask, anticancer system. Additionally, MgO NPs were chosen due to their ability to inhibit the uncontrolled growth of cancer cells, and AV was added to the NF to improve the hydrophilicity of scaffold. After blending the PLACL with the AV the contact angle was reduced from 127° to 54° due to the presence of polyphenols in AV structure.

The presence of MgO NPs causes increasing of contact angel because of the replacement of polyphenols group to MgO which is more hydrophobic. The morphology of NF scaffolds were uniform, with rough fibre surfaces. The diameter of the scaffolds was 786 ± 286, 507 ± 171, 334 ± 95, 360 ± 94 and 326 ± 80 nm for PLACL, PLACL/AV, PLACL/AV/MgO, PLACL/AV/MgO/CUR and PLACL/AV/MgO/β-CD, respectively. The obtained materials were tested on the MCF-7 cells to estimate their cytotoxicity properties by measurement the number and the shape of breast cancer cells (MCF-7). PLACL/AV/MgO/Cur and PLACL/AV/MgO/β-CD nanofibres showed the highest apoptotic effect, destroyed polygonal morphologies and reduced the number of MTC-7 cells. It is worth noticing that PLACL/AV/MgO also possessed apoptotic effect but its activity significantly increased in combination with Cur or β-CD. The obtained results showed that PLACL/AV/MgO NF scaffolds supported cell adhesion and proliferation but combination of CUR or β-CD showed only a slight cytotoxicity effect on the cell line. Moreover the reduction of the concentration of active ingredients from 5% to 1% show that the scaffold still possess anti-tumor properties. The in vitro studies showed that PLA-*co*-PCL nanofiber scaffold with natural ingredients can be effective against breast cancer cells [32].

The emulsion/solvent evaporation method is one of the common techniques to prepare polymeric microparticles (MPs) for the encapsulation of bioactive molecules [33]. The preparation of biodegradable PLA microcapsules has been extensively investigated in recent years [34,35,36]. The PLGA was used as a platform for encapsulating dexamethasone sodium phosphate (DE). DE is sensitive to degradation and requires stabilization to prolong its activity. Before encapsulation in PLGA MPs, DE was firstly physically complexed by three types of molecules: hydroxypropyl-β-CD (HPCD), γ-CD, or water-soluble polyethylenimine (PEI). The CDs were selected as entrapping agents due to their ability to form the IC, while the complexation of DE by PEI is based on electrostatic interaction between phosphoryl part of DE and nitrogen atoms of PEI. Therefore, PLGA exhibit lower encapsulation efficiency of DE, when MPs are obtained without complexion agents. The complexation of DM leads to increase encapsulation efficiency, however, PEI was found to be the most effective complexing agent since it possesses a large number of imine groups in one polymer chain. The presumptive protective effect of DE complexation by CDs and EPI against UV irradiation showed the negative result. After irritation, the free DE, as well as complexed one, underwent photodegradation to the same extent. Unexpectedly, the more stable complex between DE and γ-CD showed the highest photodegradation degree. The formation of intermolecular hydrogen bond between DE and γ-CD have destructing effect on its photochemical stability. However, the presence of any secondary interaction slows the DE release from PLGA MPs in PBS at 37 °C. Moreover, the release of DE from PLGA MPs with EPI is lower than those of the drugs from PLGA with HPCD and γ-CD. The delay of DE release from MPs with EPI is associated with stronger electrostatic interactions in comparison to weak van der Waals forces in IC [24]. PLGA microspheres were prepared also by Cannava et al. as a sustainable drug delivery system for anti-inflammatory agent Celecoxibe (CE). They evaluated the influence of IC between β-CD and CE on the size of MPs, the release of CE and anti-inflammatory activity. The presence of the IC causes the size reduction of MPs because of its ability to act as a surfactant and prevent coalescence during the evaporation process. Unexpectedly, the creation of an IC reduces the encapsulation efficiency of CE from 80% to 54%. The formation of stable IC with high solubility in water limits its ability to diffusion to the oil phase and reduce affinity to the PLGA matrix during MPs preparation. However, the formation of IC also enhances the solubility of CE in PBS thus the release of CE from all formulation with IC was much faster than from MPs without β-CD. Only 15% of CE was released from MPs without β-CD within 15 days. In addition, the presence of β-CD/CE in the PLGA matrix introduces the porosity and affects the release rate. The prepared MPs with β-CD were more effective than free drug as an anti-inflammatory drug on human chondrocyte cultures. This higher degree of activity was correlated with the better solubility of CE in the culture media and to the fact that β-CD/CE can act as a penetration enhancer [25]. The same release behaviour was observed by Gupta and co-workers for prostaglandin. The presence of IC enhances the solubility of hydrophilic prostaglandin (1.6 × 10^−2^ M at 25°C) from 10 to 60 times after addition the appropriate CDs [37], and accelerate its release and improve bioavailability. The prostaglandin administrated via pulmonary route is rapidly metabolised within 2 h at 37°C, and thus possesses short therapeutic activity. However, the encapsulation of prostaglandin into the PLGA microparticles induces the sustainable and prolonged release and thus extending the prostaglandin activity. It is well-known that all components, which build MPs are non-toxic and approved for FDA to biomedical application thus, the obtained MPs with prostaglandin were found to be safe against Calu-3 human airway epithelial cells [26].

The IC is widely used to solve the problems with solubility, degradability or bioavailability of drugs in physiological condition. The simplest method to introduce IC to polymeric NPs is based on the physical mixing of components before NPs formation [27,38]. However, the most attractive approach is to attach β-CD to the polymer chain before the formation of IC and NPs. To introduce β-CD to polymeric chain researchers used different reactions such as: azide-alkyne cycloaddition reaction (CuAAC) [39], esterification [40], carbodiimidazole coupling reaction [41] or polymerization [42]. To produce polymeric NPs with cyclodextrin moiety, Gao et al. used the well-known reaction between the carboxyl group of PLA [43,44] or PLGA with amino terminated β-CD [45]. This multistep process leads to introducing to the polymeric chain one or two β-CD moieties and obtaining the polymers with the ability to form IC. In addition, the hydrophobicity of polymers decreases as a result of attachment of β-CD moieties to the PLA chain and decreases with increasing concentration of β-CD or decreasing the molecular weight of the polymer. The NPs were fabricated by using nanoprecipitation method. The slowly instillation of acetone polymeric solution to the deionised water allows for production of spherical, uniform nanoparticles with narrow dispersity. However, the obtained NPs size and stability strongly depend on the molecular weight of polymer, solution viscosity, hydrophilic-lipophilic balance, added surfactant or encapsulated drug. The employed strategy allowed to use these nanoparticles to deliver BSA protein. The encapsulation efficiency (EE) of polymeric NPs with β-CD moiety was much higher than for non-functionalized NPs. The high EE is associated with the three types of phenomena: the creation of IC between BSA and β-CD cavity, ionic interaction of BSA carboxyl groups with protonated –NH– group of modified β-CD or amphiphilic nature of β-CD-terminated PLA. Moreover, the method used allows to control the amount of encapsulated drug. In double emulsion method, the water solution of BSA is used as an inner water phase and thus, BSA could be effectively encapsulated inside the NPs [45]. However, the influence of β-CD in the polymer chain on release behavior was not observed for all prepared NPs. As suggested by Gao et al. the degree of degradation of the polymer matrix may be responsible for the observed differences in the course of drug release curves [43,44,45]. To introduce more than two cyclodextrin moieties to the polymer, the 4-arms PEG-(PLA)_4_ β-CD end-capped copolymers were synthesized. It is worth noting that β-CD-functionalized PLGA can undergo self-assembly into reversible micelles in an aqueous solution. However, the presence of four β-CD could not induce higher encapsulation efficiency (EE) of BSA. The formation of the IC does not determine the encapsulation process and only occurs when the acrylic part of the BSA chain penetrates the cyclodextrin cavity. The encapsulation of BSA in reversible micelles is mainly based on hydrophobic interaction between PLA and BSA chain and increase with increasing of polylactide molecular weight. The release studies showed that the release of BSA from star-shaped or linear PLA-PEG NPs with β-CD moiety is faster than from PLA-PEG without β-CD. It is probably due to a faster diffusion of BSA from NPs with larger pores formed into particles in the presence of β-CD [40]. Y-shaped PDLA with β-CD moiety (β-CD-*b*-[PDLA]_2_) and Y-shaped poly(l-lactide-*co*-dimethylaminoethyl methacrylate) (PDMAEMA-*b*-[PLLA]_2_) were used to produce stereocomplexed micelles for doxorubicin (DOX) delivery. The combination of ROP, ATRP and click reaction allows for preparing this Y, unique architecture of copolymers. The presence of β-CD in copolymers allows for formation of the IC with adamantane terminated FA. Due to these untypical architectures (Y-like structure), the copolymers can create stable, uniform NPs with folic acid (FA) on the surface reinforced by stereocomplexation between enantiomeric chain of PLLA and PDLA. The cytotoxicity assay showed no obvious toxicity against human embryonic kidney cells (HEK 293 cells) no-adverse-effect and biocompatible nature of stereocomplexed micelles [46].

To prepare stereocomplex micelles another approach also was applied by Kost et al. The partly methylated-β-CD was introduced to the polymers (PLLA and PDLA) by using it as an initiator of lactides polymerization. The prepared in this way PLLA and PDLA homopolymers are still able to form host-guest inclusion complex but also stereocomplex in addition. The slow precipitation of DMF polymeric solution to distilled water allows for formation uniform, stable enantiomeric or stereocomplexed NPs. The stability of NPs in PBS at 37 °C was achieved due to the addition of poly(d,l-lactide-*co*-ethylene glycol methyl ether) to the solution during nanoprecipitation. Cholesteryl-terminated PLLA-PEG or PDLA copolymers and poly(cyclodextrin) (PCD) were used for preparation three polyester platforms containing different amount of PCD moieties for DOX delivery. The linear PCD was prepared from β-CD by using epichlorohydrin (EPI) as a linking agent [47]. The host-guest interaction between cholesterol groups and PCD based on weak van der Waals interaction and stereocomplexation are crucial interactions that led to the formation of PDLA/PLLA/PCD cross-linked micelles (CSMs). A careful selection of components allows for preparation uniform, stable dimension rage of NPs from 60 to 100 nm. Moreover, the creation of IC and increasing of PLA/PCD ratio (PLA: PCD, 1:2) improved the compactness and stability of NPs in water making it appropriate vehicle for DOX delivery A slight decrease in the DOX release profile from crosslinked stereocomplex micelles (CSMs) was observed as compared to stereocomplex micelles without PCD. The IC between PCD and cholesterol-terminated PLA forms an extra barrier, which could slow down the release of DOX from NPs under physiological condition. The cellular-uptake efficiency showed that the presence of the highest amount of PCD in CSMs causes the highest release ratio of DOX. The PCD promote the drug delivery carries to swell, dissolve and faster release of DOX from CSMs with the highest degree of PCD. The half-maximal inhibitory concentration for CSMs with the highest degree of PCD, showed the most effective proliferation activity. However, no increased toxicity was observed for all DOX loaded micelles compared to free DOX [48].

Hydroxyapatite (HA) could also be coated by PCD in a simple reaction with citric acid. This modification leads to introducing PLA to HA via ROP of LA and thus the materials composed of HA/PCD/PLA may be potentially used for bone tissue engineering. It is well-known that toxicity of materials based on HA might be correlated with Ca^2+^ release from HA. The release of calcium ions disrupts the body’s calcium homeostasis and may be the main cause of its toxicity. On the other hand, PLA chains form a hydrophobic layer on the HA surface and inhibit the free release of Ca^2+^ into the human body during application. It was also reported that the β-CD could complex some active lipophilic compounds (cholesterin) and ensure its prolonged activity. In addition, the introduction of HA/PCD/PLA into mesenchymal stem cells induces a local anabolic reaction and significantly increases its cell adhesion, mineralization, biocompatibility, and osteoinductive activity. The combination of HA/PCD/PLA reduces the cytotoxicity of HA and makes it promising material for tissue engineering [49]. The pH-sensitive micelles based on associated/disassociated IC between β-CD-terminated PLA and benzimidazole (BM) terminated-PEG were successfully synthesized as shown in Figure 3a and classic micelles, independently synthesized, based on PLA-*b*-PEG copolymer were used in the control test. The change in pH value from 5.5 to 7.4 allows for controlling the size and stability of NPs due to associated/disassociated of IC. The low pH leads to the creation of an anionic form of BM and causes dissociation of IC thereby the pH-responsive micelles become larger and more unstable. The release studies showed that by pH adjustment, 3 times faster DOX release could be achieved in pH 5.5 in comparison to the physiological condition. Furthermore, the in vitro studies were investigated on two cells line: human breast cancer cell line (HeLa) and human liver cancer cell line (HepG2). It was not observed the significant difference in the antitumor activity of pH-responsive micelles, PLA-*b*-PEG micelles and free DOX. However, all DOX-loaded micelles showed lower drug accumulation in the livers and kidney in comparison to free DOX. This results suggested that DOX-loaded micelles slightly enhance the antitumor activity and reduce systemic toxicity of DOX [29]. Additionally, the improving of targeting properties of polymeric micelles with β-CD might be achieved by conjugating folic acid to the polymer chain. The FA-modified micelles exhibit better targeting properties and could specifically delivery encapsulated drug to the tumor side due to the ability of FA to bind to folate receptors over-expressed in the cells [28,41].

The biodegradable and biocompatible PLA materials are widely used for the preparation of different types of drug carriers in the form of NP, MPs or fibres. Unfortunately, the blank polymers and their copolymers usually do not have appropriate functions enabling their direct application as drug carriers. Only the desired modifications provide them with new functions, structures and morphology allowing for controlled drug release under external stimuli. The α-, β- or γ-CD are extensively applied to improve the application properties of the drug, especially solubility in water or their penetration through cellular membranes. Although, mixing polymers with CDs is still the simplest method to improve the functionality of the polymer this operation does not always affect the drug release behavior. A promising approach to creating useful drug delivery systems is based on covalent incorporation of CDs moiety to the polymer structure by means of well-described organic reactions. It should be mentioned, however, that some of the potential drug carriers presented in the literature are very complicated in synthesis and the sophisticated materials produced in this way are usually very expensive. This limits their future use. 

## 3. Polymeric Drug Delivery Systems from Cyclodextrin and Poly(ε-Caprolactone) 

**Figure 5:**
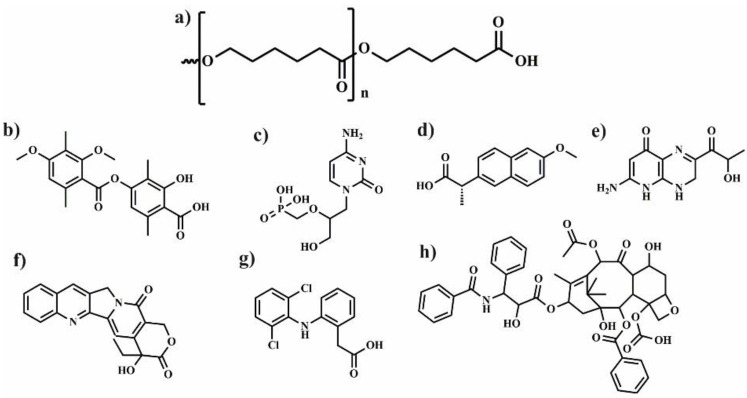
The chemical structure of (**a**) the carrier polymer -PCL, and chemical structure of (**b**) diffractaic acid, (**c**) cidofovir, (**d**) naproxen, (**e**) sepiapterin, (**f**) camptothecin, (**g**) diclofenac, (**h**) paclitaxel.

Aliphatic semi-crystalline poly(ε-caprolactone) (PCL) (Figure 5) is approved by the U.S. Food and Drug Administration (FDA) for biomedical and pharmaceutical applications. It possesses good biocompatibility and slow biodegradability, high flexibility, as well as good solubility in many the organic solvents. Therefore, PCL is a polymer frequently used as a matrix for preparation of the biodegradable and bioresorbable materials. For instance, PCL based systems were applied in the production of wound dressings, surgical sutures, prosthetics materials, as well as carriers for a variety of drugs [50,51,52,53]. However, due to the hydrophobicity and lack of active sites (similar as in PLA) needed to attach bioactive molecules, its biological applications are limited. Among the methods used to modify PCL and expanding its applications, the following strategies have been proposed: chemical modification of PCL and surface modification its particles [54,55] the formation of self-assembling copolymers with hydrophilic polymers [56,57], and coating of PCL nanofibers with bioactive materials [58,59]. The alternative way of bioactive sites addition is the preparation of non-covalent ICs of PCL with CDs (α, β, or γ-CD) [60]. Recently, PCL/CD-based systems in the form of micro- [61,62] and nanoparticles [63], micelles [64,65,66], nanofibres [67,68] or hydrogels [64,69,70] are promising drug delivery carriers due to their unique properties in terms of drug release and/or stimuli-responsiveness [64] as shown in Table 2. Therefore, in this part of the review, the application of polymeric drug delivery systems based on the combination of PCL or its copolymers and CD, are described.

Biodegradable drug-loaded polymeric microparticles (MPs) (e.g., microspheres or microcapsules) based on aliphatic polyesters are interesting and promising carriers for developing an oral-dosed controlled release [94]. The bioactive particles encapsulated in polymeric MP are usually obtained by emulsion-solvent evaporation [95]. For instance, Silva et al. reported the incorporation of CD/diffractaic acid ICs into PCL microspheres using the multiple W/O/W emulsion-solvent evaporation technique [61]. As an active substance, the diffractaic acid was used, which is known from its antiulcerogenic and gastroprotective properties. The 35-fold increase in solubility of used biologically active compound was achieved by the application of 2-hydroxy-propyl-β-CD and the formation of ICs between this drug and CD cavity. The formation of ICs during the PCL-microparticles precipitation reduced drug cytotoxicity against Vero cells, what increased safety and therapeutic efficacy. A novel class of β-CD and pH-sensitive polymer-based polymersome MPs, designed for potential therapeutic tools in the treatment of cholesterol-associated neurodegenerative diseases, was presented by the Yagci group [62]. Polymer structure was specifically designed for lysosomal-targeting through the incorporation of a benzoic imine bond between the β-CD and the PCL backbone. At physiological conditions (PBS, pH = 7.4) the obtained polymer self-assembled into stable polymersomes with a detachable β-CD core and negatively charged surface due to the presence of carboxylic-groups incorporated into PCL macromolecules. Under weakly acidic pH conditions (pH 5.5), the formed nanostructure undergoes hydrolysis of the imine linkage, with consequent disassembling and release of monomeric CDs. The obtained MPs were non-toxic according to the cellular viability test on the HUVECs line. This was a promising therapeutic approach for CD-PMs delivery to endosome and lysosome, due to rapid hydrolysis at pH 5.5 and fast cellular uptake. Besides, the presence of the carboxylic groups at the polymer termini provided the possibility of further surface modification of the microparticles for improved delivery.

The nanosized-based carriers are extensively studied as promising tools for drug delivery systems which can be passively accumulated at tumor sites due to the enhanced permeability and retention effect (EPR effect) [96]. The incorporation of amphiphilic CDs into polymers can alter the amphiphilicity of the polymer, resulting in the formation of supramolecular nanostructures self-assembled as polyrotaxanes (PRXs). For instance, CDs and PCL are able to form of PRXs structures in a solid-state, termed as “molecular necklaces”, what induces the crystallization of CDs in channel-like structures and keeps CD molecules threaded onto the polymer backbone during the post-functionalization step [97,98]. The polyester-based PRXs are described in the literature as drug delivery systems with drugs loaded by physical entrapment via hydrophobic interactions, or with drugs conjugated to the backbone of PRXs via stimulus-responsive conjugation. For example, Li et al. developed α-CD-based nanoscale micelles of PRXs structure with enzyme-regulated release behavior [63]. The novel supramolecular micelles were loaded with the anticancer camptothecin (CPT) chemically bonded with PCL through a hydrolyzable linkage. The steady-state release of drug was observed without lipase, it was shown that less than 30% CPT released over 120 h, with the negligible initial burst release. On the contrary, 50% CPT was released with 5 U (μmol/min) of lipase and 70% was released with 10 U of lipase within 10 h. To evaluate the drug encapsulation capacity of the drug in the formed micelles, DOX, another hydrophobic anticancer drug, was used as a model. DOX was loaded by dialysis method with a drug entrapment efficiency of 43.7% and drug loading efficiency of 17.3%. The release of the physically loaded DOX was compared with that of chemically conjugated camptothecin. The DOX was released much faster through diffusion, than CPT through hydrolysis of the ester bond. The enzyme-induced drug release behavior and cytotoxicity against HepG2 cells were evaluated, confirming the utility of obtained micelles for controlled drug delivery.

The unique advantages of materials obtained by combining electrospinning of aliphatic polyesters and enriching them in CDs have been discovered in the last decade [67]. For example, the nanofibers capable of capturing small molecules, such as environmental toxins in water and air, as well as hydrophilic drugs, can be obtained by this approach. The electrospinning of the aliphatic polyesters combined with CDs leads to the formation of nanofibers with improved hydrophilicity and/or crystallinity. Narayanan group first reported the successful fabrication of PCL nanofibers containing α- and γ-CDs [99], as well as β-CDs [100]. Those PCL-based nanofibers containing α- or γ-CD were prepared using electrospinning from 60:40 chloroform/*N,N*-dimethylformamide [99]. The average diameter of the obtained nanofibers increased only slightly with increasing loading of CD. However, a significant reduction in water contact angle was observed even with additions of a small percentage of CD (~5%). The phenolphthalein absorption tests showed that γ-CD-functionalized nanofibers absorbed faster than α-CD-functionalized at all CD loadings. This indicated that γ-CD was more available in unthreaded form than α-CD in their PCL nanowebs. Narayanan et al. obtained also the PCL/β-CD functional nanofibers in a similar way [100]. The efficiency of wound odor absorbance by prepared nanofibers was studied using a simulated wound odor solution, consisting of butyric and propionic acids in ethanol. Immersion tests indicated that the nanofibers containing β-CDs were very efficient in masking the odor.

**Figure 6:**
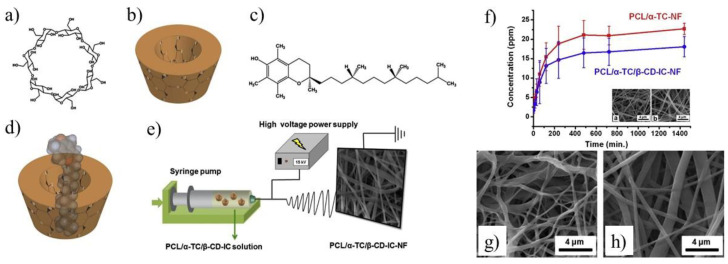
α-TC enriched PCL nanofibers preparation: chemical structure of β-CD (**a**), schematic representation of β-CD (**b**), the chemical structure of α-TC (**c**), the formation of α-TC/β-CD-IC (**d**), electrospinning of nanofibers from PCL/α-TC/β-CD-IC solution (**e**), the release of α-TC from PCL/α-TC-nanofibers and PCL/α-TC/β-CD-IC-nanofibers (**f**), SEM images of UV-treated (**g**) PCL/α -TC-NF and (**h**) PCL/α-TC/b-CD-IC-NF [71]. (Copyrights 2016 Elsevier).

In addition, the CD enriched PCL fibers were used for encapsulation of small molecules like α-tocopherol [71], naproxen [72], sulfisoxazole [68], ciprofloxacin [73], as well as even large molecule (e.g., polymer or enzyme) [74,75,98,101,102]. Those studies were initiated to enhance the stability of active compounds against various environmental factors during delivery by entrapment them in PCL nanofibers. For example, the Uyar group has chosen α-tocopherol (TC), a form of vitamin-E frequently applied as a healing factor in wound dressings, and used it as an active agent for the formation of β-CD-ICs encapsulated into electrospun nanofibers (Figure 6a–e) [71]. The marginal increase in fiber diameter (345 ± 140 nm vs. 205 ± 115 nm) was observed for PCL/α-TC nanofibers after β-CD application. An increase in the released amount of α-TC was found when it was delivered from PCL/α-TC/β-CD-IC-nanofibers, in comparison to PCL/α-TC nanofibers (Figure 6f) The SEM images of UV-treated PCL/α-TC nanofibers and PCL/α-TC/β-CD-IC-nanofibres presented in Figure 6g,h showed that nanofibers maintained their fibrous structure up to 65 min under the applied conditions. Authors proved that inclusion complexation between α-TC and β-CD during the formation of PCL electrospun nanofibers improves its photostability (~6% higher than un-encapsulated form) and antioxidant activity.

Moreover, wound dressing material based on β-CD and PCL was also obtained by Souza et al. [76]. In this work, the bioactive electrospun fibers containing silver sulfadiazine complexed with β-CD in the PCL nanofibers matrix were synthesized to modulate the drug release as well as to reduce the direct contact between silver and skin. Although complexation promoted a decrease in hemolytic index and slowed drug release, no negative effect on antimicrobial activity was observed. Among the drugs incorporated into PCL nanofibers, naproxen, and sulfisoxazole can also form the ICs with CDs, and these hydrophobic drugs are widely used for relieving pain. The Uyar group encapsulated naproxen in β-CD-cavities and embedded in a PCL nanofibres matrix [72]. Observation by means the XRD technique proved the successful incorporation of naproxen (NAP)/β-CD-complex into electrospun PCL nanofibers. The SEM imaging of the electrospun PCL/NAP and PCL/NAP/β-CD-complex nanofibers showed that the average diameter of the nanofibers was around 300 nm. In addition, the aggregates of β-CD-IC nanofibers were also observed. HPLC analysis revealed that the β-CD-complex based nanofibres releases NAP two times higher than PCL nanofibers with neat NAP. This is a very promising result for the future of drug delivery systems. Uyar group also described sulfisoxazole/hydroxypropyl-β-CD inclusion complex, incorporated in hydroxypropyl cellulose nanofibers via electrospinning [68]. Sandwich configurations were prepared by placing IC-enriched cellulose nanofibers between electrospun PCL nanofibers. As a result, PCL-nanofibers enriched structures exhibited a slower release of sulfisoxazole as compared with neat PCL-nanofibers. Tetracycline, a biocidal drug with poor water solubility, was also embedded in PCL nanofibers and encapsulated in β-CD cavities to be used in the regeneration of periodontal ligaments [77]. An antimicrobial diffusion test was performed for a set of nanofibers with the microorganisms like *Aggregatibacter actinomycetemcomitans* (*A.a*.) and *Porphyromonas gingivalis* (*P.g*.). Tests revealed significantly higher halos of bacterial inhibition against both oral bacteria in PCL nanofibers containing tetracycline/β-CD (34 ± 3 and 30 ± 3 mm for *A.a*. and *P.g*. respectively), compared to PCL nanofibers with non-complexed tetracycline (28 ± 4 and 26 ± 3 mm). The collected data indicated that nanofibers containing tetracycline/β-CD promote the adhesion and slower dentine demineralization enhance the potential of this formulation for clinical application. 

The hydrophobic biocidal drug, ciprofloxacin was combined with PCL nanofiber. The preparation of supramolecular CD/PCL containers allowed for efficient encapsulation of ciprofloxacin and use in this form The antibacterial application of free drug is impaired due to poor solubility and limited stability [73]. α-CD/ciprofloxacin and β-CD/ciprofloxacin ICs formation was carried out under two different conditions: at room temperature or with sonic energy. Larger amount of ciprofloxacin was trapped in the CDs cavity when ultra-sonication was applied. SEM analysis indicated that the incorporation of CDs/ciprofloxacin ICs inside PCL nanofibers did not affect the morphology of electrospun nanofibers. After incorporation of the ICs into PCL nanofibers, the release of ciprofloxacin was followed at pH 7.2. The release of ciprofloxacin from PCL nanofibers increased with increasing solubility of the drug via ICs formation. The drug release from nanofibers was mainly controlled by the diffusion, and this process did not affect nanofiber structures. The increase in the released amount of drug was found in stimulated physiologic environment, when it was delivered from β-CD/PCL nanofibers, compared to α-CD/PCL nanofibers. The key advantage of CDs is their facile capability to form ICs not only with small but also with large molecules. The enzymes are a type of large biomacromolecules incorporated into CD enriched PCL fibers. The introduction of CD-ICs into electrospun nanofibers having high surface area and highly porous nanostructure make them suitable substrate for biocatalyst immobilization [74,75]. For instance, catalase, an anti-free radical enzyme, was successfully immobilized onto poly(ethylene oxide) nanofibers containing γ-CDs, sandwiched between PCL nanofibers [74]. The positive influence of CDs on enzyme activity and the stability of the catalase was showed. Similar to the catalase enzyme, laccase was immobilized on γ-CD/PCL nanofibers and showed higher catalytic activity (96.48 U/mg), compared to enzymes immobilized on PCL nanofibers without CDs (23.2 U/mg) or γ-CD/laccase physical mixtures in PCL nanofibers (71.6 U/mg) [75]. During the formation of the CD-enzyme complex, the enzyme used did not lose activity and no denaturation was observed.

The core-shell nanocarriers based on amphiphilic copolymers have attracted great attention as a potential agents for cancer chemotherapy since the hydrophilic shell can ensure prolonged circulation of the carrier in blood, whereas the hydrophobic core can enhance drug loading efficiency [103,104]. Core-shell NPs from aliphatic polyesters are extensively used as promising materials for drug and gene delivery due to its useful properties, biocompatibility and excellent degradability [105,106]. Polymeric micelles made of amphiphilic copolymers, such as poly (ε-caprolactone)-block-poly (ethylene glycol) (PCL-*b*-PEG), are of great interest, especially in recent years [107]. The combination of CD-based ICs with PCL/PEG copolymers of various microstructure was widely used to solve the problem with solubility, degradability, or bioavailability of many important drugs. For instance, Varan et al. designed hydroxypropyl-β-CD coated and docetaxel-loaded nanoparticles composed of PCL and PCL-*b*-PEG to be applied as implants to site following after surgical operation of tumor [78]. The coating with CD significantly increased the drug encapsulation and anticancer efficacy against MCF-7 human breast adenocarcinoma cell lines, however, it did not change particle size and polydispersity. Those PEG-*b*-PCL and hydroxypropyl-β-CD-based systems were used farther in inkjet printing of antiviral/anticancer combination dosage forms. As a result of that research, a combination product consisting of anticancer paclitaxel and antiviral cidofovir drugs was manufactured as an adhesive film for local treatment of cervical cancers [79]. Characterization studies of obtained material showed that the printing process did not influence neither the structure of nanoparticles nor inclusion complex. The paclitaxel and cidofovir containing ink and film formulations have higher anticancer efficacy as compared with drugs solution. Incorporation of paclitaxel into PCL/PEG copolymer nanoparticles was also reported by Ahmed et al. [80]. In their report, the β-CD grafted poly(acrylic acid) was synthesized by radical polymerization, and then embedded on the surface of PCL-*b*-PEG-*b*-PCL nanoparticles through host-guest interaction and hydrogen bonding between the oxygen atom of PEG and hydrogen atom of carboxyl group of poly(acrylic acid). Paclitaxel was released smoothly without remarkable initial burst release during the in vitro drug release experiments (i.e., only 20% drug was released in the first 12h). After drug loading, the NPs displayed significant cytotoxicity against HepG2 cells. Kuplennik et al. applied 2,3,6-triacetyl-β-cyclodextrin within methoxy-PEG-*b*-PCL nanoparticles to improve the encapsulation efficiency of sepiapterin, the natural precursor of the essential cofactor tetrahydrobiopterin [81]. For this purpose, sepiapterin/cyclodextrin complexes were produced by spray-drying of binary solutions in ethanol and encapsulated within methoxy-PEG-*b*-PCL nanoparticles by nano-precipitation. The encapsulation efficiency and drug loading were 85% and 2.6%, respectively, as opposed to the much lower values (14% and 0.6%, respectively) achieved with pristine drug. Moreover, the sustained release of the sepiapterin from nanoparticles was observed, with a relatively low burst effect of 20%.

The CDs enriched core-shell nanoparticles reveal the ability for drug delivery through the skin by enhancing solubilization of lipophilic drugs as well as increasing the amount of solubilized species at the absorption site by promoting drug transport through passive diffusion [108]. The drug carrier system designed for the delivery of lipophilic drug through the skin was described by the Quaglia group. For this purpose, the core-shell nanoparticles based on PEG-*b*-PCL associated with 2-hydroxypropyl-β-CD were employed [109]. The NPs entrapping the second generation of photosensitizer Zn^2+^ phthalocyanine (ZnPc), highly lipophilic and fluorescent model molecule, were formed. The transport of ZnPc through porcine ear skin was evaluated on Franz-type diffusion cells. The confocal Raman spectroscopy demonstrated that 2-hydroxypropyl-β-CD caused an alteration of water profile in the skin and a high reduction in the degree of hydration at stratum corneum/viable epidermis interface which can promote NPs transport.

To enhance the controlled release of anticancer drugs and minimize the side effects of those drugs, the rational approach is to use stimuli-responsive PCL/PEG/CD micelles which show the response to pH [82], temperature change [110], light [65], ionic strength or enzymes. The pH-sensitive polymeric micelles, stable at the physiological pH, which can dissociate to release drugs in the acidic environment of solid tumor tissues, are playing an important role in controlled cancer treatment [111]. In this regard, the pH-sensitive PCL-*b*-PEG micelles containing a polymeric form of β-cyclodextrin were also used by Gao et al. as a copolymer block [82]. The complex micelles were formed via host-guest interactions between poly(β-CD) in diblock PEG-*b*-PCD copolymer and BM groups in BM-PCL. The DOX encapsulation efficiency of complexed micelles was up to 74.77%. The release of DOX from PEG-*b*-PCD/BM-PCL polymeric micelles was suppressed at neutral pH solutions and accelerated at acidic solutions or high temperatures. The cumulative release of DOX increased from 70.3% to 98.6% with a decrease of pH from 7.0 to 2.0. At weakly acidic conditions (pH 5.2), BM groups were protonated resulting in the partial disruption of complexation with β-CD in PEG-*b*-PCD, so the drug molecules could be slowly released from micelles, and the cumulative release reached approximately 80%.

**Figure 7:**
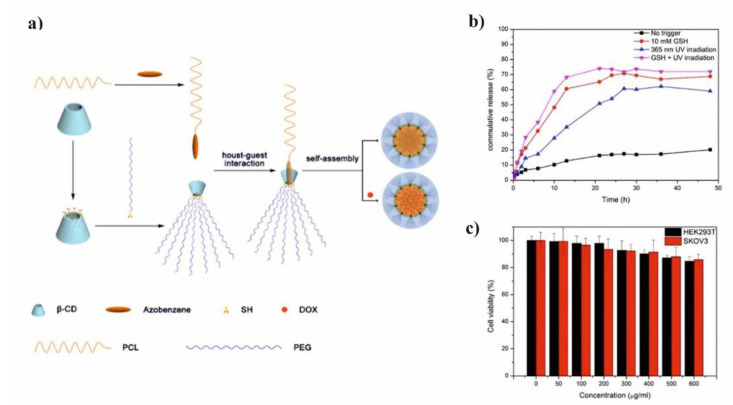
Synthesis route of glutathione/light dual-responsive supramolecular DOX loaded PCL-b-PEG-based carriers (**a**) drug release curves in different stimulations (**b**) cell viability of SKOV3, and HEK293T cells following incubation with as-prepared carriers for 24 h (**c**) [65]. (Copyrights 2019 Elsevier).

Li et al. presented glutathione (GSH)/light dual-responsive supramolecular drug carriers based on the CD modified by PEG and azobenzene-PCL fabricated for intracellular delivery of DOX (Figure 7a) [65]. The azobenzene groups/CD complexes are typical supramolecular assemblies and have been intensively studied for their unique photo-responsive properties induced by the photochemical trans-cis isomerization of the azobenzene units. The obtained spherical carriers exhibited glutathione sensitivity attributed to disulfide bonds between PEG and β-CD, and the light sensitivity response was achieved by a simple host-guest interaction between β-CD with azobenzene groups. The DOX was selected to evaluate the drug loading capacity and therapeutic effect of the carriers. The total drug loading was determined as 30.4% by UV. After 48 h of drug release experiment, the cumulative drug release rate was less than 20% without stimulation. When a stimulus (365 nm light wavelength or 10 mM GSH) or double stimulus (365 nm light wavelength and 10 mM GSH) were applied, the drug release rate was significantly accelerated, the cumulative drug release rate reached more than 55% after 48 h of drug release experiment (Figure 7b). The cytotoxicity of ICs and drug-loaded carriers were explored against normal cells (HEK293T cells) and tumor cells (SKOV3 cells). According to cytotoxicity studies of blank drug carriers, the cell viabilities were more than 85% at a wide range of concentration (0–600 mg·mL^−1^) indicating that the drug carriers exhibit lower cytotoxicity and good biocompatibility (Figure 7c). It was shown that the drug-loaded carriers have a better pharmacodynamics performance to tumor cells, but less toxicity to normal cells, since the DOX can be released under the trigger of light and glutathione. 

Star-shaped copolymers attracted much attention because their branched structures can form unimolecular micelles with better stability than the micelles self-assembled from conventional linear copolymers [112]. It is well known that those copolymers provide a stable environment for drug loading and its sustained release [113,114]. Those particular features made amphiphilic star-shaped copolymers especially useful for the formation of drug delivery systems, e.g., supramolecular NPs or hydrogel preparation. To simplify the preparation of star-shaped structures with a precisely controlled degree of branching, the supramolecular host-guest pair can be used as the block junction. For instance, Gou et al. reported the synthesis of novel drug-conjugated amphiphilic PCL star copolymers containing β-CD as core moiety. In this work, PCL/poly(acrylic acid) [115] and PCL/PEG multimiktoarm [116] copolymers were synthesized by the combination of controlled ring-opening polymerization with “click” chemistry and atom transfer radical polymerization, respectively. These new types of amphiphilic copolymers, which were composed of biocompatible poly(acrylic acid) or PEG corona surrounding both biodegradable CD core and PCL arms could self-assemble into multimorphological aggregates in aqueous solution. In addition, the hydrophobic ibuprofen-loaded nanoparticles fabricated from these drug-conjugated PCL/PEG/CD copolymers were investigated [116]. The hydrophobic ibuprofen was incorporated into the chain ends of the PCL by the reaction in the presence of dicyclohexylcarbodiimide and 4-dimethylaminopyridine. The drug-loading efficiency and drug-encapsulation efficiency of the ibuprofen-conjugated miktoarm copolymers were significantly higher than those of the corresponding non-drug conjugated counterpart. It was a result of the conjugated ibuprofen influence on the hydrophobicity of the miktoarm star-shaped copolymer, leading to an increase of drug loading amount, as well as interactions (such as *π*-*π* aromatic stacking force) between covalently bonded ibuprofen with the free ibuprofen, which forces the free ibuprofen to incorporate into the micellar core.

DOX loaded nanocarriers based on the star-shaped amphiphilic mPEG-*b*-PCL copolymers and β-CD were described by Li et al. [83]. Authors developed smart, reductive stimulus-responsive nanosystems using modified β-CD molecules. The secondary hydroxyl groups of CD were methylated to improve solubility, whereas the primary hydroxyl groups were conjugated with mPEG-*b*-PCL-SH through a disulfide linkage to amplify the hydrophobic cavity and enhance the stability of the nanocarriers. The DOX-loaded micelles were prepared with the highest drug loading capacity (LC) of 31.9 wt.% and encapsulation efficiency (EE) of 83.9%. DOX release from the micelles under a reductive stimulus was carried out in PBS (pH 7.4) and in 1,4-dithio-threitol solution which simulated the reductive tumor microenvironment. DOX was released significantly faster in the presence of DTT than in its absence, and the cumulative release rate of DOX loaded micelles containing methylated β-CD in the DTT solution was > 50% within 8 h. In contrast, the cumulative release rate in PBS was < 20% even after 100 h. It confirmed the usability of disulfide bonds, which can rapidly be broken under reducing conditions and the accelerated macromolecules dissociation is observed. It was shown that synthesized nanocarriers accumulated at the tumor site via EPR and released the drug in a controlled manner in the reductive tumor microenvironment, with negligible premature leakage, and side effects on the healthy tissues.

**Figure 8:**
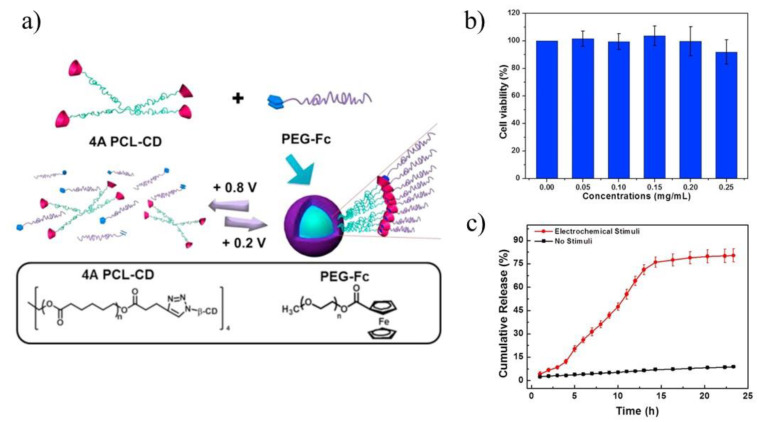
Structures of 4-arm PCL-CD and PEG-Fc and schematic representation of the potential-responsive controlled assembly and disassembly of the 4-arm PCL-CD/Fc-PEG micelles (**a**), cytotoxicity evaluation of the 4-arm PCL-CD/Fc-PEG micellar solutions, measured by comparing the cell viability of A549 cells (**b**), the dependence of the amount of DOX released from 4-arm PCL-CD/Fc-PEG micelles and time under no stimuli and +0.8 V stimuli (**c**) [84]. (Copyright 2019 Elsevier).

The release of drugs from the star-shaped copolymeric micelles can be also induced by the reactive oxygen species. The example of those carriers are systems with oxidation-sensitiveness due to non-covalent, combination of β-CD/ferrocene. Those systems are able to produce an excess amount of reactive oxygen species in the specific tumor cell lines [117]. In this regard, the electrochemical redox stimulus systems based on β-CDand ferrocene linker were described by Yuan [84] and Wei [85] groups. Yuan and co-workers applied 4-arms PCL terminated with β-CD and linear polymer polyethylene glycol terminated with ferrocene to improve the biocompatibility and efficiency of DOX delivery (Figure 8a) [84]. The electrochemically-responsive supramolecular micelles were obtained, which exhibited faster release and better biocompatibility compared with their linear analogues, namely linear ferrocene-terminated PEGs (Fc-PEGs). The cyclic voltammetry and 2D NOE NMR were used to confirm the host-guest interaction between these two polymers. Cytotoxicity experiment of the supramolecular micelles, conducted on A549 cells, proved their biocompatibility (Figure 8b). Through electrochemical control, a reversible assembly-disassembly transition of the micelles was realized, which was investigated by TEM. In order to confirm the high efficiency of star polymers as drug carriers, UV-vis spectra were used to calculate the drug loading content and drug loading efficiency respectively, which turned out to be 11.0% and 67.7% (8.0% and 49.4% in case of linear analogues). In vitro drug release experiments under electrochemical stimuli showed that DOX could be released from drug carriers in several hours (Figure 8c). Upon applying a potential of +0.8 V, DOX was released, however, along with the decomposition of micelles caused by the oxidation of ferrocene attached with PEG macromolecules. Those comparative studies revealed the advantages in drug loading of the star-shaped copolymers over linear analogues for use as drug carriers.

In addition, the Wei group described the supramolecular structures obtained from 3, 4, and 6 arm star-shaped PCLs with ferrocene end-capped arms and 3-arm poly(oligo ethylene glycol) methacrylates terminated by β-CD [85]. The micelles obtained from star-shaped exhibited the highest drug loading content and the encapsulation efficiency, most likely due to its highest stability reflected by its critical aggregation concentration value. The in vitro drug release profiles, at the physiological conditions (PBS, pH = 7.4) and in an oxidizing medium (PBS, pH = 7.4, 0.2 mM NaClO) at 37 °C, proved that NaClO significantly promoted the drug release with 20–30% increase for all formulations, confirming the oxidation-triggered dissociation of β-CD/Fc complexation and the structural deformation of supramolecular micelles. Finally, the cytotoxicity tests of all supramolecular star-shaped micelle constructs for HeLa cells, revealed that DOX-loaded micelle formulations exhibit lower cytotoxic activity than the free DOX, most likely due to the slower internalization mechanism (endocytosis vs direct membrane permeation). The cytotoxicity studies proved also that higher degree branching of the obtained star-shaped copolymer and growing hydrophilic arm lengths enhanced the therapeutic efficacy of the DOX-loaded nanocarriers. 

The PCL/PEG copolymers and CD-based supramolecular hydrogels (SMGels) with their reversible sol-gel transition properties were widely explored as injectable biomaterials capable of establishing versatile drug delivery systems [70,86,118]. The SMGel based on α-CD [87,119], β-CD [64], or γ-CD [86] and PCL/PEG copolymers of various microstructure were developed and investigated due to their controlled drug release and site-specific drug delivery triggered by various stimuli. The advantage of SMGel is the dynamic nature of their structure that can be easily broken by shear forces because it is composed of weak noncovalent interaction. Tabassi et al. presented the example of SMGel with shear-thinning thixotropic behaviour, showing that hydrogel composed of copolymers with a PCL to PEG ratio of 1:4 are suitable for syringeable SMGel preparation [87]. The mixing of α-CD (12%) and PCL-*b*-PEG-*b*-PCL (10%) induces gel formation in less than one minute and enables sustained release of vitamin B12 for at least 20 days. Moreover, their thixotropic behavior makes supramolecular hydrogels highly attractive for many biomedical applications, e.g., ocular drug delivery. Zhang et al. were the first who reported thixotropic SMGel based on α-CD and a low-molecular-weight mPEG/PCL block copolymer for ocular drug encapsulation [69]. The SMGel containing diclofenac, known as an anti-inflammatory drug, showed relatively low cytotoxicity toward L-929 and HCEC cells. The hydrogel was nonirritant toward the rabbit eyes, what was confirmed by the Draize test, fluorescein staining, as well as histological observation. The application of Nile Red-labeled micellar supramolecular hydrogel proved that it significantly extends the retention time on the rabbit’s corneal surface compared with a plain micellar formulation. 

A variety of active substances were introduced to the PCL/PEG copolymer-based SMGels with pseudo-polyrotaxane structures formed by CD and PEG blocks. For example, by mixing PEG-*b*-PCL micelles that solubilize DOX, poly(ethylene glycol)-*b*-poly(acrylic acid) (PEG-*b*-PAA) micelles that host cisplatin, together with α-CD, results in the preparation of a dual-drug loaded pPR based hydrogel [88]. The erosion of the gels resulted in a discrete release of micelles from which the drugs were delivered. In vitro cytotoxicity studies proved that DOX-loaded hydrogel inhibited the growth of human bladder carcinoma EJ cells, whereas the dual drug-loaded SMGel showed significantly higher cytotoxicity against applied cells. The thixotropic and injectable SMGel based on PPRXs formation were described also by Xu et al. [66]. Authors confirmed that hydrophobic cores formed by self-assembly of amphiphilic polymer methoxy-poly(ethylene glycol)-*b*-poly(ε-caprolactone-*co*-1,4,8-trioxa[4.6]spiro-9-undecanone copolymer and the microcrystals of PRXs formed by α-CD and PEG blocks could serve as two-level cross-linking for the gel formation. The in vitro and in vivo degradation demonstrated the general release of NPs, which can be easily uptaken by cells and accumulate at the tumor site. Further studies on the paclitaxel controlled release and antitumor efficiency were also performed [89]. Most importantly, the obtained hydrogel was efficient in inhibiting tumor cells growth and prevented the diffusion of paclitaxel to other mice tissues.

Besides the small molecules, also proteins and genes were encapsulated and released from SMGels. The injectable and thixotropic SMGels of α-CD with methoxyPEG-poly(ε-caprolactone)-(dodecanedioic acid)-poly(ε-caprolactone)-methoxyPEG triblock polymer (α-CD/mPEG-*b*-PCL-*b*-mPEG) were proposed for sustained release of recombinant human erythropoietin (rhEPO) in an acute myocardial infarction rat model [90]. The rapid gelation of this system enabled effective encapsulation of rhEPO at the injection site, which improved cardiac function for 30 days after myocardial infarction and allows for avoidance of polycythaemia, a well-known collateral effect of rhEPO. Khodaverdi et al. reported **γ**-CD in preparation of an insulin-loaded supramolecular PCL-*b*-PEG-*b*-PCL based gel, with low hemolytic activity and superior biodegradability compared to those prepared with α-CD [86]. In this system, aggregations of γ-CD threading onto PEG blocks were supported by a small number of hydrophobic PCL blocks and a high number of hydrophilic blocks. The SMGels were obtained by mixing 10.5% (*w/v*) γ-CD and 2.5% (*w/v*) copolymer and revealed an excellent syringeability. Insulin was released up to 80% over 20 days, keeping its initial folding.

On the other hand, the methoxyPEG-poly(ε-caprolactone)-poly[2-(dimethylamino)ethyl methacrylate] triblock polymer (mPEG-*b*-PCL-*b*-PDMAEMA) and α-CD were used to form stable polyplexes with plasmid DNA (pDNA) [91]. The pDNA was electrostatically bonded to the cationic segment of the copolymer. The mPEG-PCL-PDMAEMA copolymers exhibit a good ability to condense pDNA into 275−405 nm polyplexes with hydrophilic mPEG in the outer corona. The multiple mPEG chains were used as cross-linking moieties to anchor the DNA nanoparticles within the α-CD/PEG supramolecular PPRXs hydrogel system. The obtained hydrogels revealed controlled release for several days without detrimental effects on protein expression level. In vitro gene transfection results showed that the supernatants containing pDNA released from the hydrogels at various time points had good bioactivity. However, in vitro cytotoxicity of copolymers assay on COS7 cells confirmed that PDMAEMA chains length has a significant impact on the biocompatibility of the whole copolymers.

The SMGels revealing thermosensitivity, able to form an injectable solution at low temperatures and non-flowing gel at around physiological body temperature, were also extensively studied. The thermosensitive gels based on PCL/PEG copolymer and CD-ICs were presented in the literature as a promising systems for non-steroidal anti-inflammatory drug delivery [64,92]. A novel injectable, in situ gel-forming drug delivery system based on thermosensitive β-CD-modified PCL-*b*-PEG-*b*-PCL was studied by Wei et al. [64]. The applied copolymer can self-assemble in water to form a micelle solution, with a sol-gel transition occurring as the temperature increased, which was confirmed to be related to the polymer concentration. The linkage of β-CD to the hydrophobic macromolecule chain ends made the encapsulation of hydrophobic drug within the hydrogel networks more effective. Subsequently, the in vitro release behavior of indomethacin from the micelles was investigated. According to the cumulative release profile, the drug was sustainably released up to 50% over 9 days.

Indomethacin was released without remarkable initial burst release and the release behavior followed a linear course for 48 h, indicating that the obtained micelles could be applied as a depot for drug-controlled release. Additionally, two in vivo models, i.e., carrageenan-induced acute arthritis and Freund’s complete adjuvant-induced arthritis were employed to evaluate the therapeutic effect of the drug after subcutaneous administration in the right-back paw of rats. A significant improvement in the anti-inflammatory effect of indomethacin in rats occurred after encapsulation in the obtained hydrogel network. Khodaverdi investigated thermosensitive PCL-*b*-PEG-*b*-PCL based SMGel obtained by IC with γ-CD as a carrier for sustained release of dexamethasone [92]. The SMGel with excellent syringeability was prepared by mixing 20 wt.% γ-CD and 10 wt.% of the copolymer in a few seconds. It is worth noting that the applied solution of the synthesized copolymer, with a PCL/PEG ratio of 1/5, could turn into a gel only in the presence of γ-CD due to the short PCL blocks and insufficient hydrophobic interactions between polymer chains. The rheological studies revealed the shear-thinning behavior of the obtained SMGel. The release profiles showed that formulation containing 0.1% dexamethasone released about 40–46% of the loaded drug after 23 days. It was shown that the release of dexamethasone from the supramolecular gel occurred slowly, with a slight initial burst release.

**Figure 9:**
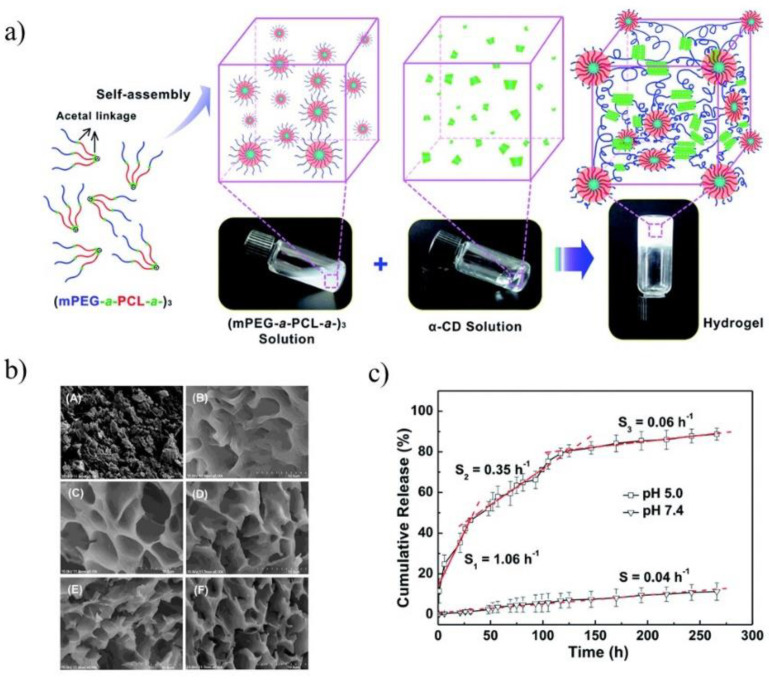
Illustration of acid-cleavable hydrogel networks based on inclusion complexes between (mPEG_45_-acetal-PCL_27_-acetal-)_3_ and α-CD (**a**), SEM images of lyophilized hydrogels from copolymers varied with the content of hydrophilic and hydrophobic segments (**b**), (in vitro cumulative release of encapsulated DOX·HCl from (mPEG_45_-acetal-PCL_27_-acetal-)_3_ hydrogel at 37 °C under different conditions: pH 5.0 buffer solution and pH 7.4 buffer solution (**c**) [70]. (Copyrights, 2016 The Royal Society of Chemistry).

The pH-sensitive SMGels, formed within a few minutes in an aqueous medium, are investigated as potential “smart” drug delivery carriers. Hu et al. developed an injectable hydrogel based on inclusion complexes of the star-block copolymer from mPEG and PCL linked with acid-cleavable acetal groups ((mPEG-acetal-PCL-acetal)_3_) (Figure 9a) [70]. The ICs aggregated into necklace-like crystalline PRXs and acted as physical crosslinking joints for the hydrogels, while the remaining uncovered hydrophilic PEG chains functioned as water-absorbing segments. The obtained SMGels revealed unique structure-related reversible gel-sol transition properties at a certain level of stress. Importantly, according to SEM observation, the lyophilized hydrogels exhibited a porous sponge-like structure and could be used as drug delivery depots (Figure 9b). In vitro drug release test showed that encapsulated DOX was released from the drug-loaded hydrogels in a controlled and pH-dependent manner (Figure 9c).

In this part of the review, the versatility of CD/PCL based supramolecular structures were presented, clearly demonstrating the suitability of IC assemblies in the form of nanocarriers or colloid-associated gels for diverse therapeutic demands. In all cases, the CD application resulted in improved delivery of hydrophobic drugs. The CD-ICs containing small bioactive molecules or even large molecules (e.g., enzymes) were designed and used for tailor/modulate release from multilayer nanofibrous structures or core-shell structures based on PCL and its copolymers. The necklace-like PRXs structures, stable due to hydrophobic and van der Waals interactions between the inner surface of the CDs and the PCL chains, were found as particularly useful for encapsulation/grafting of the biomolecules. However, both IC capability, as well as functionalization of hydroxyl groups present on external rims of CD and PCL macromolecules, are still not fully explored. The highly organized ICs based drug delivery systems capable of responding to external or internal stimuli, such as temperature, pH, light, or redox alterations, are presented as examples of useful carriers for several diseases treatment providing excellent extracellular stability and effective intracellular drug release. Nevertheless, it is anticipated that the studies of multi-stimuli responsive PCl/ICs structures will be continued and provide advanced carriers able to overcome both extra- and intracellular barriers. Although various successful in vitro and in vivo studies demonstrating pharmacologic, antimicrobial, and antioxidant effects are reported, the lack of comprehensive evaluation of systemic toxicology as well as biodegradability of CD-based supramolecular drug delivery systems opens a field for futher studies.

## 4. Poly(Ethylene Glycol)/Cyclodextrin Systems for Biomedical Applications

**Figure 10:**
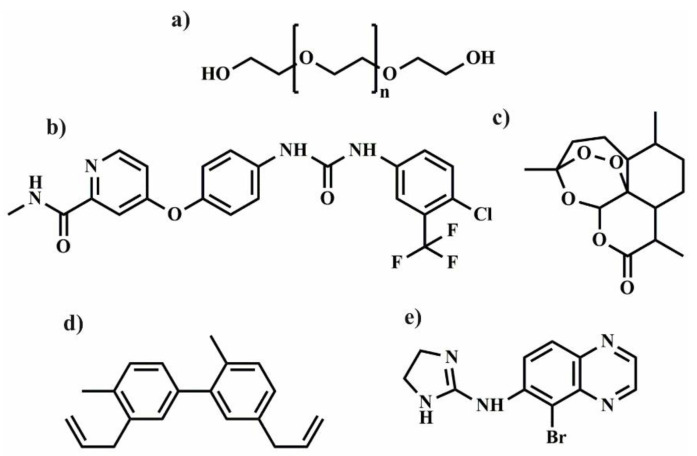
The chemical structure of (**a**) the carrier polymer-PEG, and chemical structure of (**b**) sorafenib, (**c**) artemisinin, (**d**) honokiol, (**e**) brimonidine.

PEG (Figure 10) is a gold standard typically used to modify biomacromolecules and synthetic macromolecules, that is subsequently applied to prepare the drug carriers with improved physicochemical properties and circulation in the body [120] The first example of this type modification was shown in pioneering work from the late 1970s, where PEGylation provided protection for proteins against destruction during their administration [121]. Currently, the term PEGylation is associated with the covalent coupling of PEG with biological and synthetic molecules [122]. Since CDs are typically used to host the drug molecules in their interior, the PEGylation was a rational strategy to additionally improve their biodistribution in vivo. The combination of PEG and CDs is frequently used for the preparation of NPs, micelles, gels and hydrogels (Table 3). In this respect, Rojas-Aguirre et al. [123] proposed using click chemistry to combine β-CD with different molar mass PEG chains (5000, 2000, and 550 g/mol) and prepared star-shaped PEGylated β-cyclodextrin. Copper(I)-catalysed azide−alkyne cycloaddition (CuAAC) [124] between alkyne modified PEG and azide-functionalized CDs, was also employed, however, the mixture of products with different level of substitution was obtained. Subsequently, the synthesized star-shaped PEGylated β-CD was tested against human monocytes, Vero and HeLa cells, and after incubation no effect on the viability of most cells was observed with except, however, for β-CD-PEG550, which reduced the viability of HeLa cells and human monocytes. The observed effect was ascribed to the PEG molecular weight and architecture of PEGylated β-CD. Although, the copper was extracted during the synthesis, its content level in the final products was not investigated. Since copper may affect the toxicity [125] residual traces may influence the observed results. The most often described literature examples concerns the interactions between CDs and adamantyl (Ada)-functionalized, water-soluble polymers [126]. Using this approach, the dextran (DXT) was modified with β-CD, Ada, and PEG-Ada/PEG-CD, in which PEG macromolecules were used as a flexible spacer. After simple mixing, the nanoassemblies were spontaneously formed in water. The presence of PEG spacer leads to the formation of less compact and smaller nanoparticles due to the higher binding constant of guest polymer (DXT-gPEG-Ada) in relation to the DTX-Ada. However, the opposite relation was observed for DXT-gPEG-CD/DTX-CD for which the presence of spacer decreases decreased the binding constant. These results implicate that by the careful adjusting of the binding constant of host-guest interactions between macromolecules, the control over the structure of resulting nanoassemblies could be achieved [127].

Polyrotaxanes (PRXs) and polypseudorotaxanes (PPRXs) are different examples of host-guest interlocked complexes in which linear molecule or polymer are encircled by macrocyclic components (e.g., CDs) [128]. The influence of the formation of PPRXs during the microparticles (MPs) preparation was investigated for the system composed of CDs and PEG [129]. The emulsifying process, using polypropylene glycol (PPG) as an oil phase and CDs with and without PEG as a water phase, was used to prepare water-in-oil (W/O) emulsion. The solidification into desired MPs occurs during the lowering of the temperature.

The authors claim that the irregular MPs morphology was observed due to the formation of CD/PEG PPRX, whereas the regular structure of MPs was formed for the CD/PPG PPRX. Moreover, α-CD was essential for the formation of PPRXs, as the complex was not formed for β- and γ-CDs. The strategy based on the application of polyrotaxanes for the construction of drug delivery carriers was proposed also by Moon et al. [130]. The described preparation required a four-step process which consists of (1) inclusion complexation of β-CDs with amine-terminated PEG, (2) the blocking of PEG end groups with l-tyrosine (l-Tyr), and (3) modification of β-CDs by succinic anhydride, to formed PRXs for DOX delivery. The opened succinic anhydride were applied, to attach the DOX to the PRXs to induce control release of DOX from PRXs by cleavage the hydrolyzable ester bond. To prove this concept, the in vitro release experiment in the phosphate buffer saline was performed. For the first 48 h, the zero-order kinetics without significant burst release was observed. The control release was also observed for PPRXs micelles composed of β-CDs and PEG terminated with protoporphyrin (PpIX) [131]. The PpIX-functionalized PEG by a transformation of its hydroxyl end groups into the amine end groups, and, subsequently coupling carboxylic functionalities in PpIX were performed. The micelles (MCs) were prepared by simply mixing both components in water and CMC value was 12 μg/mL, as a consequence spherical nanoparticles with a diameter less than 100 nm in size were obtained. The formation of PPRXs in the MCs was verified by XRD where two types of the structure dominate: head to head or tail to tail tunnel structure. As a further step, three different DOX-loaded micelles were prepared in which PPRXs differs with the number of β-CDs (PPRX-2, PPRX-9, PPRX-13). It was observed that with the increasing number of β-CDs in PPRXs the size of MCs increased whereas the drug loading content decreased. Moreover, the stability and size of the MCs composed PPRX-2 and PPRX-9 was invariant after DOX encapsulation, however, the MCs sizes from PPRX-13 increased in time. As a result, the release of DOX from these nanoparticles depends on the ratio of PEG to PPRX in the resulting nanocarrier. Both in pH = 5.0 and pH = 7.0, the fastest DOX release for PPRX-13 MCs was observed, and it was attributed to the enhanced swelling of nanocarriers with the increase of the β-CDs number and lower π-π conjugation level of DOX with PpIX. Subsequently, HepG2 cell lines (human liver cancer) were chosen to test the anticancer activity, IC_50_ values were again the lowest for PPRX-13 MCs what reflects to the DOX release rate. In contrary, the cellular uptake was the highest for PPRX-2 MCs due to their smallest size ~45 nm in comparison to the PPRX-9 MCs (~75 nm) and PPRX-13 MCs (~89–150 nm).

**Table 3:** Summary of described hydrogels, micelles and nanoparticles for drug delivery based on combination of PEG and its copolymers with different CDs.

Type of Drug Delivery System	Platform	Type of CD	Drug	Release Medium	In Vitro/In Vivo Studies	Ref.
Micelles	PpIX-PEG	α-CDs	DOX	buffer (pH = 5.0 or pH = 7.4)	HepG2 cells	[131]
Nanoparticles	Star-shaped polymers CD-g-TPGS with different TPGS	β-CDs	DOX	PBS	MCF7 and ADR/MCF7 Cells/H22 sarcoma model	[132]
Nanoparticles	Folic acid–poly-ethylene glycol–β-cyclodextrin (FA–PEG–β-CD)	β-CDs	DOX	PBS (pH = 5.0 or pH = 7.4)	HepG2 cells	[133]
Nanoparticles	CDPF consisting of β-CD, PEG, and FA	β-CDs	DOX	PBS (pH 5.5, 6.8 and 7.4)	MCF7 cells/ Male NCRNU nude mice	[134]
Micelles	Ferrocene conjugated PEG (PEG-Fc) and β-CD-hydrazone-DOX	β-CDs	DOX	PBS (pH = 5.0 or pH = 7.4)	HeLa cells	[135]
Nanoparticles	PEG-HPG-BM and FA-CD	β-CDs	DOX	PBS (pH 5.3, 6.8 or 7.4)	HeLa, HepG2, L929 cells	[136]
Nanoparticles	PEG-CD/AD/SF	β-CDs	DOX sorafenib	PBS (pH = 5.0 or pH = 7.4)	HepG2 cells	[137]
Nanospheres	DMPE-mPEG2000/γ-CD-C10	γ-CDs	artemisinin	-	Intravenously injection to Wistar rats	[138]
Micelles	F127-CD conjugate	β-CDs	honokiol	PBS (pH = 7.4)	*Candida albicans* as test strain	[139]
Nanoparticles	PEI-CD·PEG-AD·FA-AD	β-CDs	pDNA	-	FR-negative HEK293 and FR-positive KB cells	[140]
Nanoparticles	FA-PEG-GUG-β-CDE/DOX/ siPLK1	β-CDs	siRNA/DOX	PBS (pH 7.4) or citrate buffer (pH 5.5)	KB cells and BALB/c nu/nu mice	[141]
Micelles	P(Asp-co-AspGA)/P(Asp-co-AspPBA)	α-CDs	vancomycin	PBS (pH = 7.4)	-	[142]
Nanoparticles	PNSC@APEG	β-CDs	5-FU	PBS (pH = 7.4)	NIH3T3 cell lines	[143]
Nanoparticles	PNS-SS-A CD-HPEG	β-CDs	DOX	PBS (pH = 7.4)	HeLa cells	[144]
Nanoparticles	β-CD-PEG capped ZnO	β-CDs	Cur	PBS (pH 7.4) or acetate buffer (pH = 4.8)	MCF7 cells	[145]
Gel	β-CD/PEG	β-CDs	diclofenac	PBS (pH = 7.4)/pig skins	-	[146]
Hydrogel	α-CD/PEO–PHB–PEO	α-CDs	dextran-FITC	PBS	-	[147]
Hydrogel	β-CD-NCO/ NH_2_-PEG-NH_2_	β-CDs	lysozyme, β-estradiol quinine	PBS (pH = 7.2)	-	[148]
Hydrogel	α-CD/ 4-arm-PEG	α-CDs	brimonidine	PBS	-	[149]
Hydrogel	α-CD/ A-PEG-A T-PEG-T	α-CDs	DOX	PBS (pH = 7.4)	L929 cells/Sprague Dawley (SD) rats/Chinese Kunming (KM) female mice	[150]
Hydrogel	PEG-β-CyD/PEG-Ad	β-CDs	Tf-AF647	PBS	HeLa cells	[151]
Hydrogel	β-CD/ Pluronic^®^ 127	β-CDs	curcumin	PBS (pH = 7.4)/acidic buffer solution (pH 1.2)	HeLa, MCF-7 and L929 cells	[152]
Hydrogel	α-CD/ NPOD-PEG	α-CDs	DOX	PBS (pH 7.4) or acetate buffer (pH 5.0)	A549 cells	[153]

**Figure 11:**
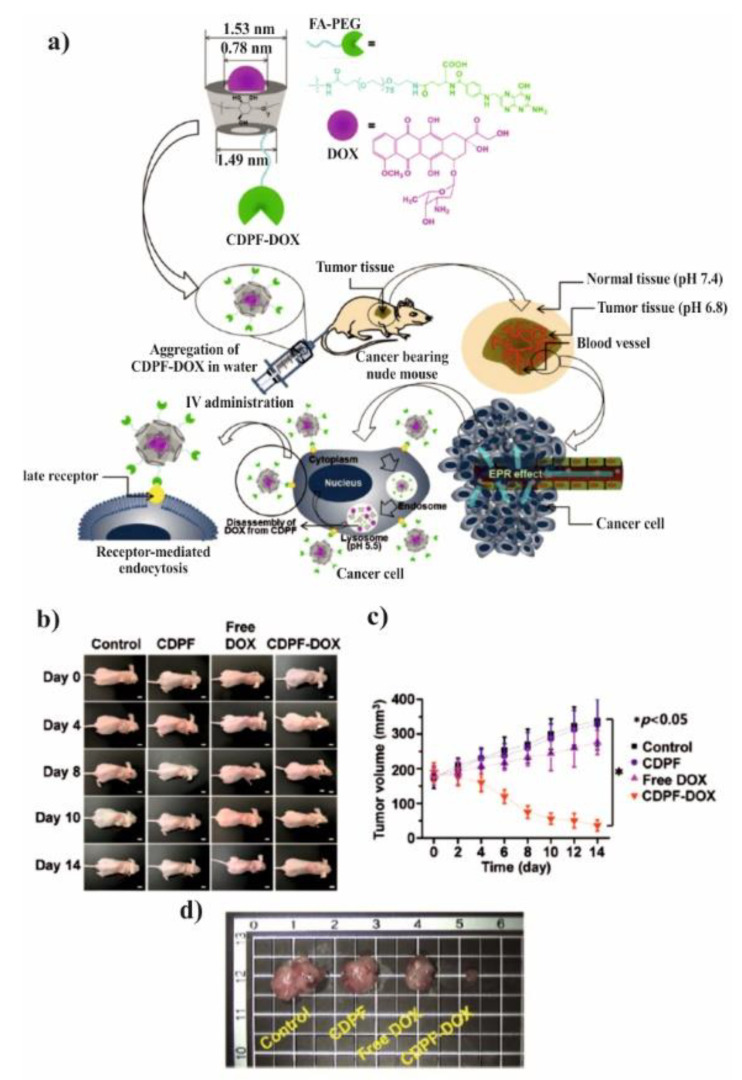
(**a**) Inclusion complex between FA–PEG–β-CD and DOX and schematic illustration of the endocytosis of FA–PEG–β-CD-DOX into cancer cells. (**b**) Gross appearances of tumor tissues in control, CDPF, free DOX _HCl and CDPF-DOX treated mice observed at 0, 4, 8, 10 and 14 days. (**c**) Tumor volume (mm3) and of mice treated with the samples at 0, 2, 4, 6, 8, 10, 12 and 14 days. Free DOX HCl and CDPF-DOX were intravenously injected via the lateral tail vein on days 1 and 8. Error bars represent mean SD (n = 5); the measurement of the cancer volume was repeated three times (* *p* < 0.05 compared with control). (**d**) Tumor images. (Copyright 2019 Elsevier).

Nowadays, there is a growing interest in the application of host-guest interactions of CDs/drugs with a combination of hydrophilic PEG macromolecules in the preparation of polymeric nanoassemblies for DOX delivery. Both the covalent conjugation [132,133,134] and supramolecular complexation [135,136,137] were used. The first strategy was focused on the conjugation of targeting molecules along with hydrophilic PEG macromolecules applying two types of targeting molecules: folic acid (FA) and d-α-tocopheryl (α-TC). The FA is a vitamin which exhibits remarkable tumor targeting ability because it is overexpressed on the surfaces of a variety of human cancers such as breast, nasopharyngeal, cervical, ovarian, and colorectal cancers [154]. Therefore, folic acid–polyethylene glycol–β-cyclodextrin (FA–PEG–β-CD) was prepared to improve DOX delivery to targeted lines human liver cancer cells (HepG2) [133] and breast cancer (MCF-7) [134], as shown in Figure 11. The desired FA–PEG–β-CD was obtained by the reaction of carboxyl-functionalized FA-PEG-COOH [133] or amine-functionalized FA-PEG-NH_2_ with β-CD [134].

The spherical DOX-loaded nanoparticles with the diameter ranging from 40 to 55 nm were obtained by simple mixing both components in water and purified by dialysis. The obtained NPs show the pH-dependent release of DOX and higher release in pH 5.5 in comparison to pH 7.4, 7.2, and 6.8, which was attributed to the protonation/deprotonation of β-CD and DOX in acidic conditions. For the HepG2 cells, it was shown that the DOX was delivered successfully to cells, however, there was no comparison to the free drug. In contrary, it was done for MCF-7 cells and their viability was significantly reduced after incubation with FA–PEG–β-CD (CDPF) and DOX as compared to free DOX. This indicates that the inclusion complex formation between β-CD and DOX enhanced the cytotoxicity rate towards cancer cells. To prove this concept, the in vivo experiments were performed and after intravenous injection of CDPF/DOX the tumour volume was lower in comparison to control, CDPF, and free DOX, as shown in Figure 11a–c. Moreover, CDPF/DOX treatment induced the overall necrosis cancer tissue, not only partial as for free DOX, as shown in Figure 11d. Thus, it could be concluded that the FA–PEG–β-CD/DOX drug delivery system can effectively deliver the DOX to the tumour tissue and decrease its side effects.

In addition, d-α-tocopheryl polyethylene glycol succinate (TPGS) can be also used as targeting moiety against different tumours to enhance intracellular drug concentration which, as a result, increases the efficiency of the therapy [155,156]. It was found that one of the major mechanism of multidrug resistance in cancer cells is enhanced drug efflux by energy-dependent pump [157]. For instance, P-glycoprotein (P-gp) is responsible for such efflux, since it is overexpressed in many cancer cells. It was a rational strategy to combine CDs with TPGS because both are the P-gp inhibitors. Therefore, the multistep reactions were employed for the synthesis of CD-TPGS conjugates with a different number of arms (CD-2TPGS-2arms, CD-4TPGS-4arms, CD-6TPGS-6arms) in which the final step was the condensation of hydrazine-functionalized β-CD and aldehyde group of TPGS (prepared after its reactions with 4-formylbenzoic acid). Subsequently, the nanoparticles with the size ranging from 207.5 to 222.3 nm were obtained by a solvent evaporation method, however those with two TPGS were unstable, therefore the CD-4TPGS and CD-6TPGS were further investigated. The drug loading (41.3, 43.5%) and release (74.7, 76%) was similar for both formulations, DOX/CD-6TPGS and DOX/CD-4TPGS, respectively. The DOX-loaded nanoparticles composed of CD-4TPGS and CD-6TPGS showed similar cytotoxicity against MCF-7 cells to the free DOX, however great supremacy of cytotoxicity against MCF7/ADR drug-resistant cancer cell in comparison to free drug. Subsequently, in vivo antitumor effect was tested on the H22 sarcoma model, and the tumour inhibition was similar for CD-4TPGS and CD-6TPGS and slightly better than free DOX. This result was consistent with the cellular uptake and in vitro cytotoxicity. Moreover, NPs can be safely used as a drug delivery system, since the test of hepatotoxicity indicate that there was not a significant effect for different organs after their administration.

Stimuli-responsive DOX-loaded nanocarriers based on CD and PEG macromolecules were also proposed to improve the efficiency of antitumor therapeutics. In this regard, the pH-sensitive nanoparticles composed of benzimidazole end-capped poly(ethylene glycol)-hyperbranched polyglycerol (PEG-HPG-BM) and FA-modified β-CD (FA-CD) were designed for the preparation of targeted supramolecular nanoparticles (TSNs) [136]. The nanoparticles were formed by the supramolecular interactions between BM functionalities and CD due to its sensitivity to different pH values. The formed NPs (78–88 nm) showed pH-sensitive DOX-release in comparison to non-sensitive NPs (without CD-FA complexation) and this was attributed to the protonation of BM in acidic conditions. The cytotoxicity of NPs against the HeLa cells was highest for the TSNs due to the presence of FA targeting molecule. However, for the HepG2 cells for which FA is not targeting moiety, the effect of TSNs is less pronounced, therefore the combination of pH-sensitivity and targeting is required to efficiently enhance the therapy outcome. In addition, Song et al. proposed to use Ada and CD inclusion complexation for the preparation of a reduction-responsive drug delivery system for the delivery of both DOX and sorafenib (SF) [137]. To achieve this aim, the synthesis of both Ada-terminated doxorubicin prodrug with disulphide bonds and PEG-CD was performed. The NPs were prepared by inverse nanoprecipitation and after the addition of SF the size of NPs increase from 166 to 186 nm. The release of DOX and SF in pH 5 and pH 7.4 was much faster in the presence of dithiothreitol (DTT) which was a reducing agent able to cleave the disulphide bond. However, the released amounts of SF were significantly higher because it can only interact with the building blocks of NPs by hydrophobic interactions or p-p stacking, whereas DOX was connected by a chemical-reversible bond. Most importantly, the in vitro cytotoxicity essay demonstrate that PEG-CD/AD/SF NPs exhibit great efficiency against HepG2 cells in comparison to free SF and PEG-CD/AD+SF physical mixtures. Furthermore, dual responsive MCs based on β-CD-hydrazone-DOX and ferrocene-functionalized PEG (PEG-Fc) were designed. The conjugation of DOX to the CD by hydrazide groups was performed in the three-step procedure, whereas the PEG end-group modification was done by the reaction with ferrocenecarboxylic acid. The MCs were formed by spontaneous self-assembly in water and their size was ranging from 42 and 62 nm. The DOX-release profiles revealed that the DOX can be rapidly released at the acidic conditions (pH 5) and by reactive oxygen species (H_2_O_2_), typically present in cancer cells. This result was attributed to the hydrolysis of hydrazine bonds between DOX and CD, whereas the accelerated release with the addition of H_2_O_2_ was related to the oxidation of PEG-Fc and shedding of MCs shell. As a final step, it was shown that DOX was accumulated in the nucleus of HeLa cells slightly slower than the free DOX due to the fact that in the MCs the DOX should be cleaved in endosomal pH to subsequently reach the nucleus.

Apart from DOX, different kinds of drugs were encapsulated in the nanocarriers composed of CD and PEG molecules. For instance, artemisinin-loaded γ-CD NPs combined with polyethylene glycol (PEG) derivatives (polysorbate 80 and DMPE-mPEG2000) [138] and honokiol-loaded pluronic F127-cyclodextrin MCs [139] were tested in vivo. The first systems showed good activity against malaria, whereas the second one against fungal infections (*Candida albicans*). For both systems, the encapsulation of the drug into the nanocarrier could enhance the circulation time in the blood flow (longer elimination half-life) what increase the therapeutic effect of the drug. Moreover, the targeting by FA was used for the delivery of plasmid DNA (pDNA) with nanoparticles build by supramolecular complexation from Ada-functionalized FA, PEG-Ada, and β-CD-grafted with branched polyethylenimine with low molecular weight (PEI-CD). The two modes of complexation were employed in which (A) PEI-CD, FA-Ada, and PEG-Ada were complexed before the addition of pDNA, whereas in (B) firstly the pDNA was complexed with PEI-CD and subsequently the host-guest complexation was induced by the presence of FA-Ada and PEG-Ada. The aim was to obtain the polyplexes with the size in the range 50–200 nm that predispose their facile cellular uptake. The ability of each formulation to bind pDNA at various N/P ratios was examined by agarose gel electrophoresis. Method A gives rise to NPs of slightly lower size in comparison to method B, and the polyplex A was able to inhibit the DNA migration at nitrogen/phosphorus (N/P) ratios of 3, whereas for polyplex B inhibition occurs at N/P ratio of 10. However, for in vitro transfection, two polyplexes were prepared with N/P 20, naked pDNA (ND), and commercially available PEI-25KD, and tested by means of a luciferase activity assay. The activity was tested out in folate receptors (FR)-negative HEK293 and FR-positive KB cells. As expected, the effect of polyplexes was the highest for FR-positive cells, however, for both cellular models the efficiency followed the order Method A > Method B > PEI-CD. This could be attributed to the higher stability and lower size of polyplexes and as a result their better cellular internalization. It was concluded that especially polyplex A could be a good alternative for branched PEI-25KD due to lower toxicity and better transfection efficiency [140]. Furthermore, FA-targeting was used to selective, simultaneous delivery of siRNA and DOX by a ternary complex of FA-PEG-GUG-β-CDE/DOX/siPLK1. The main part of a carrier was FA-polyethylene glycol (PEG)-appended polyamidoamine (PAMAM) dendrimer (generation 3; G3) conjugated with glucuronylglucosyl-β-CyD (GUG-β-CyD). The positively charged nanoparticles with a size of 92 nm were prepared by mixing the carrier with DOX in a ratio of 1:3 and carrier to siRNA ratio of 50. It was shown that the ternary complex reaches the 94 % cytotoxicity activity in KB cells which was significantly higher than binary complex FA-PEG-GUG-β-CDE/siPLK1. Subsequently, an intravenous injection of the polyplex was administered twice a week to BALB/c nu/nu mice, whereby both the tumour size and weight were also significantly lower for FA-PEG-GUG-β-CDE/DOX/siPLK1 than the binary complex. Moreover, there were no side-effects of the therapy due to negligible mice body change after the administration. The obtained results showed great potential in a tumour selective-therapy [141]. In addition, stimuli-responsive glucose-responsive NPs were prepared by the complexation PEG-*b*-poly(aspartic acid) derivatives, such as: glucosamine (GA)-functionalized block copolymer PEG_45_-*b*-P(Asp-*co*-AspGA), phenylboronic acid grafted-block copolymers of PEG_114_-*b*-P(Asp-*co*-AspPBA) and α-CD. This sophisticated system self-assemble into core-shell (CS) micelles in PBS due to the formation of α-CD/PEG_45_ inclusion complex and GA/PBA cycloborate. Subsequently, α-CD was removed by dialysis and the desired hollow vehicles with a diameter of 40–60 nm were obtained composed of cross-linked P(Asp-*co*-AspGA)/P(Asp-*co*-AspPBA). It was shown that the nanovesicles swell in the presence of glucose and their size increases depending on the glucose concentration. Moreover, the release of vancomycin (glycopeptide antibiotic) can be also controlled by varying the type of added sugar (fructose versus glucose). The higher release of vancomycin in the presence of fructose was attributed to its higher affinity for PBA in comparison to glucose [142].

Another strategy allowing nanocarriers formulation and drug encapsulation is based on silica [143,144] or zinc oxide [145] nanoparticles surface modification with CD and PEG macromolecules. For instance, the CD was conjugated on porous nanosilica (PNS), subsequently, the supramolecular complexation with Ada-PEG was performed along with the addition of 5-fluorouracil (5-FU). The resulting 5-FU loaded NPs size was 50 nm and the release of the drug lasted for 3 days. However, although the nanocarrier possesses acceptable cytotoxicity against NIH3T3 cell lines, its efficiency was lower than the free drug [143]. In contrary, Nguyen et al. proposed to functionalize PNS with adamantylamine (A) by disulfide bonds (PNS-SS-A) which were subsequently supramolecularly complexed with cyclodextrin-heparin-polyethylene glycol (CD-HPEG) [144]. The size of nanoparticles was approximately 40–50 nm and drug encapsulation efficiency of DOX was 56%. The release of DOX could be enhanced by the presence of DTT due to the redox-sensitive dissociation of disulfide bonds present on the surface of PNS. After incubation with HeLa cells, the dose-dependent cytotoxicity was observed for nanoparticles and free DOX. However, once again the free drug and DOX loaded PNS were more efficient than the supramolecular complexed NPs. The authors ascribed this phenomenon to free DOX aqueous solubility and membrane permeability. Moreover, luminescent zinc oxide nanoparticles were coated with PEG and β-CD by wet co-precipitation method and used for curcumin (Cur) delivery. The author describes the formed NPs as oval shape carriers with visible micellar core-shell morphology and NPs diameter was in the range of 20–23 nm. The release was tested in the physiological pH of 7.4 and tumor lysosomal pH environment of 4.8 and the higher amount of the drug was released in the acidic environment. The authors concluded that the drug release profiles are following fickian diffusion in which initially the first-order kinetics is observed followed by sustained release via zero order kinetics. The advantage of application of these NPs was the strong fluorescence of zinc oxide NPs which allows for their tracking in cells and brine shrimp. Moreover, the efficiency against the Staph bacteria of PEG-β-CD coated ZnO nanoparticles was moderate, in contrary good anticancer activity against MCF-7 cells was observed, however not better than drug-loaded zinc oxide NPs without supramolecular modification [145].

Gels and hydrogels can be also proposed as drug delivery depots for the administration of drugs and proteins [158]. The gels and hydrogels are a perfect environment that protects the drug and allow for controlled diffusion of their payloads by adjusting the crosslinking density [159]. There is only one example of gels composed of the PEG and CD for the diclofenac delivery [146]. The gels were prepared with a concentration of 0.1 M β-CD and 0.54 M K_2_CO_3_ in PEG400, respectively [160]. The occurrence of the gelation was ascribed to the formation of PPRXs supramolecular structure. Two types of thermo-reversible gels were formed at the gelation temperature close to the human body. It was observed that the release of diclofenac for the obtained gels was significantly higher in comparison to commercial products. Its release rate also reflects in the skin penetration properties, after the initial lag phase, the higher flux and *K*_p_ (permeability coefficient) for the PPRXs gels indicate that the gel was suitable for dermal formulation.

A different strategy was proposed by Li et al. [147] based on inclusion complexation between a biodegradable poly(ethylene oxide)–poly[(R)-3-hydroxybutyrate]–poly(ethylene oxide) (PEO–PHB–PEO) triblock copolymer and α-CD. Due to the formation of PPRX structure between α-CD and hydrophobic interactions between the middle PHB blocks, the strong network of supramolecular hydrogels was created. Subsequently, the release of fluorescein isothiocyanate labelled dextran (dextran-FITC) from the hydrogels with different composition was investigated. The best performance was obtained for the hydrogel composed of α-CD in a high concentration (9.7 wt.%) and PEO-PHB-PEO with a molecular weight of 5000-3140-5000 g/mol, respectively. For this formulation, the sustained release lasting for one month was achieved, whereas for other formulation faster release kinetics was observed. Therefore, by the delicate tuning, both the PHB block length and inclusion between the α-CD/PEG, the optimal strength of the hydrogel can be obtained. It was concluded that such drug-loaded hydrogel can be easily injected under pressure to desired tissue due to its thixotropic properties and subsequently slowly release its cargo. Moreover, CD/PEG hydrogels were prepared by conjugation of NH_2_-PEG-NH_2_ with β-CD with isocyanate groups in DMSO and incubation in distilled water [148]. The CD was functionalized with approximately five isocyanate groups and gelation occurred immediately after the addition of amino-functionalized PEG, however to slightly slow down this process, the acetic acid was added during the formulation. The release of the three different model drugs: lysozyme, β-estradiol and quinine release, was investigated in PBS. The drug physicochemical properties strongly influence the release from CD/PEG matrix since lysozyme is a hydrophilic, quinine is also hydrophilic able to form weak inclusion complexes with CD whereas hydrophobic β-estradiol create a strong inclusion complex with CD. Moreover, the release was additionally controlled by the CD content which was in an agreement with swelling/loading experiments, because a tighter network decreases the diffusion/penetration process. In conclusion, the lysozyme was rapidly released, whereas for quinine and β-estradiol the bi-modal profiles were observed due to drug-CD inclusion complexes. Additionally, the shear stress can be used to control the release of the drug from PEG/CD hydrogels [149]. For this purpose, 4-arm-PEG were mixed α-CD to form supramolecular hydrogel with polyrotaxane structure that exhibits reversible gel-sol transition. Its structure can be easily broken by shear forces and such thixotropic behaviour allows for hydrogel injection through a syringe needle. This shear-tinning behaviour was used to control the release of brimonidine by shaking a gel with PBS solution in different rates. As could be expected, the release of the model drug was much faster under external stress due to disassembly of hydrogel supramolecular structure.

**Figure 12:**
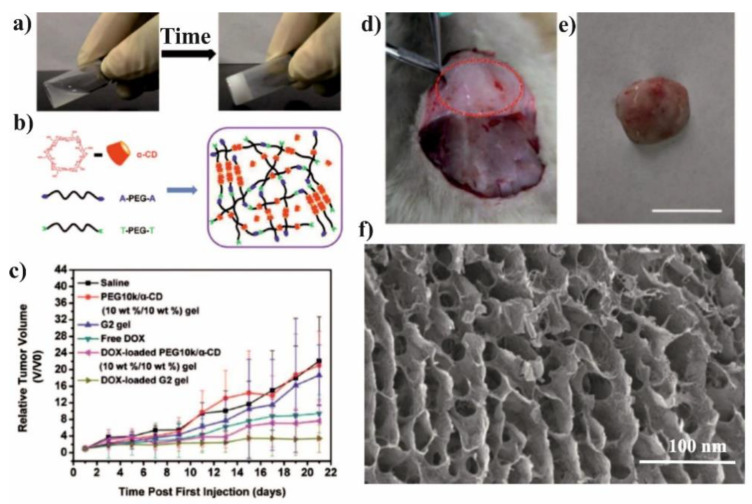
(**a**) Photographs of an A-PEG10k-A/T-PEG10k-T/α-CD aqueous solution (PEG/α-CD, 10 wt.%/10 wt.% and a G2 hydrogel. (**b**) Schematic illustration of the gelation mechanism of the supramolecular hydrogel. Changes in relative tumor volume (**c**) and in relative body weight. Each solution was injected into xenograft-bearing mice (U14) after the initial tumor volume had reached 150–250 mm^3^. In vivo formation of the G2 hydrogel in the subcutaneous tissue after 30 min (marked as red-dotted curves) (**d**), the G2 gel removed from the rat (bar = 1.5 cm) (**e**) and the corresponding SEM of the lyophilized hydrogel (bar = 100 mm) (**f**). (Copyright 2013, RSC).

Supramolecular hydrogel was also prepared by mixing adenine- and thymine-functionalized PEG (A-PEG-A/T-PEG-T) with α-CD for the delivery of DOX [150], as shown in Figure 12a,b. The presence of A and T end groups enhanced the elastic modulus, however, their influence on the drug release in different pH was notfigureinvestigated. Nevertheless, the sustained release of DOX in PBS (pH 7.4) was observed, depending on the drug loading content. Subsequently, the cytotoxicity test showed that freeze-dried powder of hydrogels is relatively safe at lower concentrations, whereas at higher concentrations the viability of L929 cells decreases due to the effect of α-CD which is toxic at the concentration of 12.5 mg/mL. Most importantly, the supramolecular hydrogel can be also formed in vivo after subcutaneous injection into SD rats by a needle, as shown in Figure 12d,e. In addition, the injected hydrogels preserved its porous structure after injection (Figure 12f) which allows for its good permeability to the biological medium. The in vivo intra-tumoral injection indicated that the A-T complexed gel (DOX-loaded G2 gel) exhibit better therapeutic effects due to restricted tumour growth in comparison to the controls and gel composed of α-CD/PEG without nucleobases (DOX-loaded PEG10k/α-CD (10 wt.%/10 wt.%) gel), as shown in Figure 12c. It was correlated to the elasticity of hydrogel containing nucleobases which lead to the longer release of DOX. Since, the application of supramolecular complexed hydrogel does not cause the reduction of the rats’ body weight, it was concluded that it is a good candidate for anti-cancer therapy in which the sustained release of the drug is required.

Hennink et al. proposed [161] the use of star-shaped 8-arm PEG (PEG8) modified either by β-CD or cholesterol moiety with a hydrolyzable ester bonds. The hydrogels were prepared in ammonium acetate buffer (pH 4.7) due to the presence of reversible supramolecular interactions (inclusion complexes CD/cholesterol), the obtained material was thermo-reversible. In the following paper, this strategy was used to investigate a degradation and protein/peptides release from supramolecular complexed polymer networks [162]. Four different model payloads were used to test the hydrogel properties as drug delivery depot: lysozyme, bovine serum albumin (BSA), and immunoglobulin G (IgG). The sustained release of loaded proteins was observed regardless of their hydrodynamic diameter, however by an increase of solid content of hydrogel from 22.5% (*w/w*) to 35% (*w/w*) prolonged release could be achieved. It was concluded that the release is mainly governed by the surface erosion of hydrogel. Moreover, it was shown for a small peptide (bradykinin) that at high concentration of hydrogel components the release of a peptide depends also on the hydrogel surface erosion, however, it follows first-order kinetics. The alternative way for achieving the controlled release of proteins from CD/PEG hydrogels is to use the ultrasounds as a trigger for their release [151]. To acquire such ultrasound-responsive structure the host-guest between adamantine-PEG and β-CD-functionalized eight-branched PEG was used. It was shown that Tf-AF647 (transferrin) can be specifically released after the ultrasound exposure at the desired part of a hydrogel due to its structure degradation. Nevertheless, the hydrogel degraded after exposure, however, it could spontaneously be re-formed after removal of stimulus, and the Tf-AF647 clearly moved to the upper part of PBS indicating its successful release. As a model of a living body, the HeLa cells with a collagen layer were used to observe the intracellular accumulation of transferrin. To induce the transport through collagen layer to the cells, the hydrogel should be exposed to the ultrasound stimulus and it could be done by a repeating pulsation which causes cleavage of host-guest binding. Therefore, this system could release the proteins in an ultrasound-guided manner.

Thermo-responsive hydrogels were prepared from β-CD/PEG mixed with poly (ethylene oxide)-poly (propylene oxide)-poly(ethylene oxide) tri-block copolymer (Pluronic^®^ 127) for the delivery of curcumin (Cur) [152]. The micellar type hydrogel with LCST characteristics was formed in which PPO was encompassed by β-CD as a center block and PEO macromolecules as the micelle shell. By heating the supramolecular hydrogel above and below LCST, the reversible gel to sol transition can be achieved. Moreover, the hydrogel exhibit much higher swelling in PBS (pH 7.4) in comparison to acidic buffer (pH 1.2), therefore the release of Cur was tested by varying both the temperature and pH. The optimum conditions to obtain the highest Cur release were pH 7.4 (at 35 °C) due to the solvent uptake which causes the hydrogel pores opening and facilitates the diffusion of the drug. A slight decrease at 40 °C was attributed to the aggregation of hydrophobic domains. In contrary, the influence of temperature in acidic medium was negligible due to the deionization of functional groups and low degree of swelling. Additionally, with increasing the concertation of PEG the release of Cur in pH 7.4 (in 35 °C) was faster, whereas increasing content P127 slow down the release of Cur at both temperatures and in both dissolution media. Subsequently, the cytocompatibility of unloaded and Cur-loaded hydrogels was tested against mouse fibroblast cell line and the obtained material exhibited good safety in comparison to commercially available Triton-X 100. After the inhibition of hydrogel with HeLa and MCF7 cells, hydrogel exhibits good efficiency in killing cancer cells, however lower than the free drug. Therefore, it can be used for storage and the sustained delivery of Cur to cancer cells. Moreover, Shi et al. proposed to use α-CD/PEG hydrogels for the co-delivery of both hydrophobic (NPOD) and hydrophilic (DOX) anticancer drugs as a consequence of acidic environment present in the tumor tissues [153]. To achieve this aim PEG (2000 g/mol) was firstly reacted with 4-formylbenzoic to obtain aldehyde-functionalized PEG which was subsequently used for the reaction with 4-aminopodophyllotoxin (NPOD). As a result, NPOD-PEG conjugate was synthesized with pH-sensitive imine bond. The XRD studies confirm the PPRX structure of the obtained hydrogel in which the α-CD rings are stacked along the NPOD-PEG chains and additionally this structure is stabilized by strong hydrogen-bond interaction between NPOD end groups. Such a structure composed of supra-cross-links exhibited the good shear-thinning behavior which is required for the hydrogel biomedical applications. Subsequently, the ability of hydrogels for the triggered, site-specific drug delivery was tested under two different pH values (pH 5.0, 7.4). The release of DOX and NPOD was relatively low (approximately 40%) at pH 7.4 while the accelerated release of DOX and steady release of NPOD was observed after decreasing the pH to 5.0. This was attributed to the fact that in pH 7.4 the release was mainly caused by diffusion whereas at pH 5.0 it was mainly controlled by a breakup of supramolecular structure. The lower release of NPOD in acidic conditions was correlated to the inclusion complex formation of PEG-NPOD with α-CD which slow down the hydrolysis. As a final step, cytotoxicity against the A549 human lung cancer cells was tested and the obtained hydrogel exhibited strong therapeutic effect in comparison to controls and free NPOD in all concentrations. This indicated the advantage of the combination of hydrophobic and hydrophilic drugs in cancer therapy with single-modality treatment.

The combination of PEG/CD in one material leads to the development of novel drug delivery systems both in the nanoscale (nanoparticles, micelles) and macroscale (gels, hydrogels). These systems were mainly used for the delivery of anticancer drugs and proteins. To enhance the therapeutic effect of encapsulated drugs targeting moieties were incorporated or a different methods of the stimulus (pH, temperature, ultrasound, shear) were used to control the release of loaded drug in the desired target. Moreover, a lot of effort has been made to test obtained systems both in vitro and in vivo. The investigated systems possess good biocompatibility and efficiency in therapy after drug encapsulation. Despite of the fact that many drug delivery systems based on CD have been reported by various research groups, those focused on the delivery of anti-cancer drugs are limited. For instance, CRLX101 polymeric nanoparticle-containing camptothecin (CPT) based on the CD/PEG conjugate was in the clinical trials [163]. Therefore, we believe that the effort of the scientist and the pharmaceutical industry should be focused on the translation of PEG/CD drug delivery systems from the laboratory to clinics.

## 5. Cyclodextrin Conjugate to Polysaccharides for Drug Delivery

**Figure 13:**
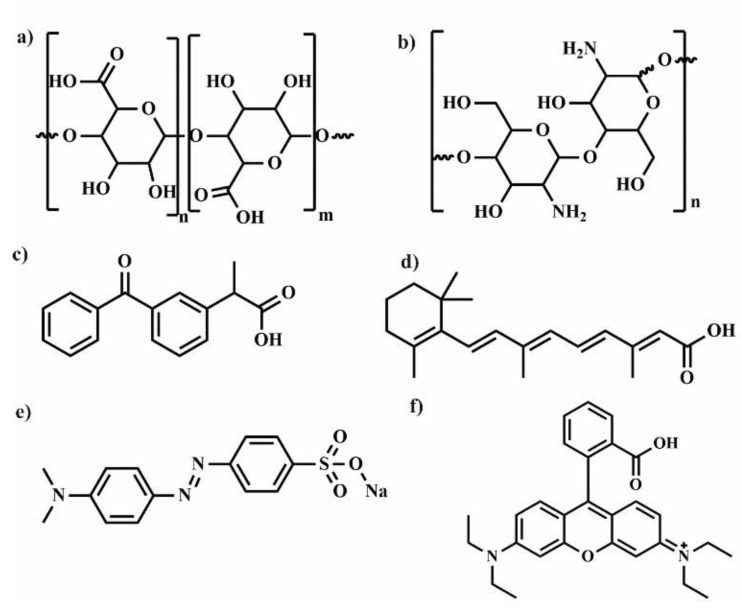
The chemical structure of the carrier polymer (**a**) alginate and (**b**) chitosan, and chemical structure of (**c**) ketprofen, (**d**) retinoic acid, (**e**) methyl orange, (**f**) rhodamine B.

Natural polysaccharides (Figure 13) are biocompatible, biodegradable, renewable linear or branched polymers consisting of different glucose units crosslinked by glycoside bonds [164,165]. These natural biomolecules have shown remarkable pharmaceutical activities such as anti-cancer activity [166], antiviral property [167] anti-inflammation activity [168] cardiovascular protection, and anti-mutation effects [169]. Moreover, the presence of many reactive groups (hydroxyl, amino, and carboxylic) allows for various chemical modification enabling, for instance, co-polymer grafting or atom transfer radical polymerization (ATRP) to improve the physical and chemical properties of polysaccharides [170].

**Figure 14:**
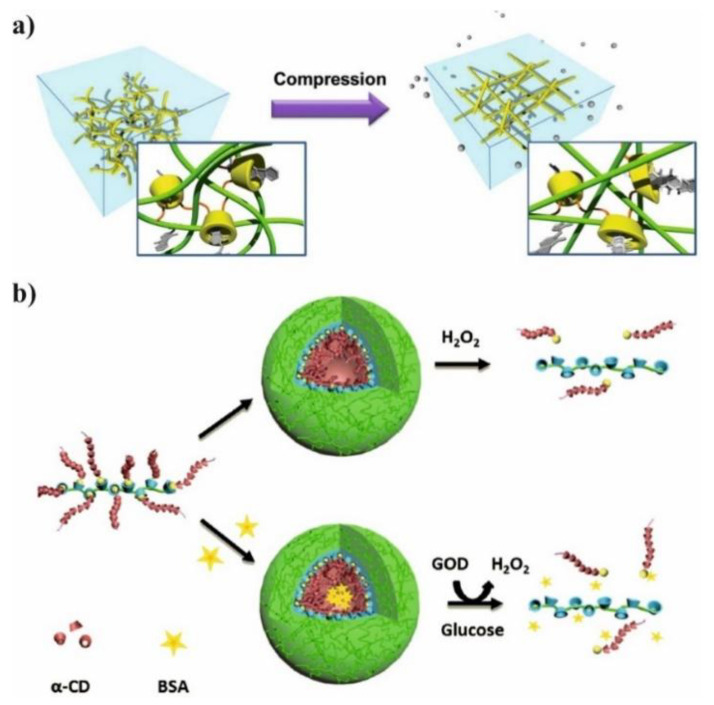
Two different approaches to controlled release: controlled release under mechanical compression (**a**) and oxidizing agents (**b**). (Copyrights 2019 Scientific report, Copyright 2018 Frontiers in Pharmacology).

β-CD is one of the most popular agents for modification of polysaccharides aimed for biomedical applications [171,172]. Therefore, the polysaccharides are excellent scaffolding material for cyclodextrin which enables for the preparation of different drug delivery systems (Table 4) with high encapsulation efficiency [173]. The crosslinking methods were used to combine β-CD with alginate gel (CCALs) for controlled drug delivery. The resulting hydrogels were obtained by simple mixing of sodium alginate (AL) with previously prepared crosslinker (β-CD activated by ethylene diamine). Next, the obtained hydrogels were immersed in CaCl_2_ solution for the replacement of Na^+^ ions for Ca^2+^. The complexation of free carboxylic groups in AL chain by Ca^2+^ additionally improve the mechanical properties of CCAL. To elucidate the release profile under the mechanical stimulus (Figure 14a), the ondansetron (ODN) was employed as a model drug. The controlled release of ODN from CCAL hydrogel can be achieved by mechanical stimuli. The faster release of ODN was observed in the case of increasing compression above 30%, due to the regulation of the host-guest interaction. The β-CD moiety is distorted as a result of applied force, which probably causes the guest to be pushed out of the cavity and thus, the release of drug become faster however it is difficult to determine the exact effect of mechanical stimuli on the polymer structure. This method could show a novel approach for controlled drug release from hydrogels in comparison to other stimuli such as heat, light, and magnetic force. In addition, the delayed release was observed as a result of the formation of IC between ODN and β-CD [174].

A similar approach for the synthesis of alginate conjugated with β-CD was applied by Achamann and co-workers [175]. Oxidation of alginate, reductive amination, and azido-alkyne cycloaddition click-reaction were employed to covalently anchor β-CD to alginate chain. Subsequently, the water solution of CaCl_2_ was mixed with a different type of alginates to form four different hydrogel beads: unmodified alginate, unmodified alginate in physical mixture with β-CD, and unmodified alginate with a mixture of alginate conjugated β-CD in two concentrations (25% and 50%). The release studies showed that hydrogel beads with β-CD chemically crosslinked to alginate are able to absorb higher amount of methyl orange and slow down its release than the other formulations. However, the obtain hydrogel beads are strongly unstable during release studies performed in 0.9% NaCl solution as a result of replacement Ca^2+^ ions for Na^+^ [175].

As an alternative to AL, dextran is a biopolymer that can be used for hydrogel preparation. Simple modification of dextran by maleic anhydride leads to the obtaining of reactive scaffold suitable for the hydrogels production [176]. The injectable hydrogels were prepared by a multi thiol-ene click reaction. Dextran modified by maleic anhydride could be easily crosslinked by applying per-6-thio-β-CD (PSCD) or di-thiolated end-capping poly(ethylene glycol) (DSPEG). After mixing of components, the microscopic hydrogels (CD-gels or CD-PEG-gels) were created via Michael addition between carbon-carbon double bond of maleic anhydride and thiol groups of PSCD or DSPEG. The obtained hydrogels were composed of particles with a diameter less than 100 nm and the obtained materials were non-toxic against zebrafish embryos. The retinoic acid (RA) was used as a simple model thus CD-gels or CD-PEG-gels loaded with RA were prepared. The presence of IC between RA and β-CD lead to control release of RA in PBS what could be correlated with in vivo release studies [176].

**Figure 15:**
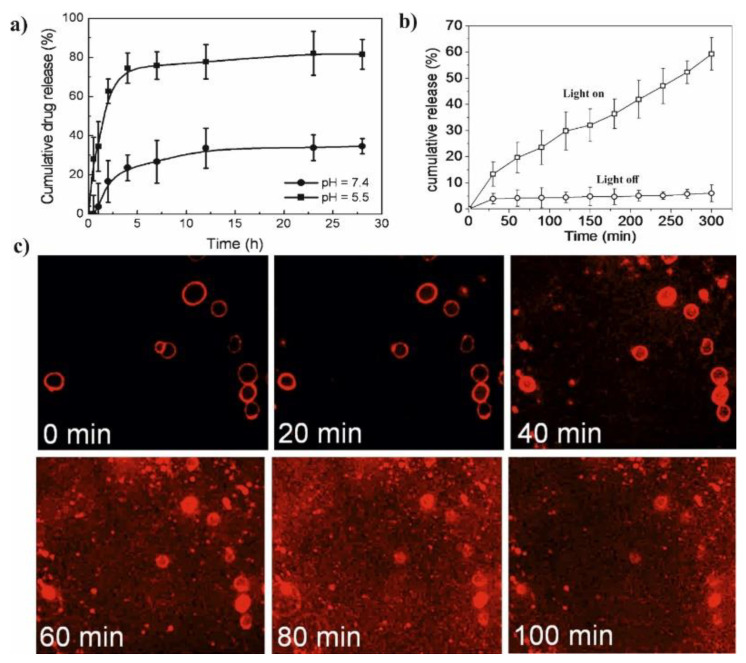
Drug release from (**a**) pH-sensitive dextran microcapsules, (**b**) from photo-switchable dextran microcapsules, and (**c**) snapshots of the photo-dissociation of photo-switchable dextran microcapsules- loaded with Rod A. (Copyrights 2011 ACS (1**a**), Copyrights 2011 ACS (1**b**)(1**c**)).

Microcapsules are a promising carriers for drug delivery due to their simple administration, the effective protection of encapsulated drugs against degradation in their interior, and as a result, the possibility to control their release over time [183]. For the controlled release of rhodamine B (Rod B), biodegradable microcapsules consisting of dextran/poly(aspartic acid) (PASP) were prepared by Zhang et al. [177]. The multistep reaction included: a) functionalization of dextran by β-CD in Schiff’s base reaction, b) preparation of PASP with adamantane backbones, c) deposition of both polymers on CaCO_3_ modified by polycations surface, and finally d) removal of CaCO_3_ by EDTA, which allows for obtaining pH-responsive hollow microcapsules for Rod B delivery. The stability of microcapsules is based on the IC formation between β-CD grafted to dextran and adamantane group anchored to PASP. Moreover, the Schiff base bond between β-CD and dextran (-C=N-) could be easily hydrolysed in a weakly acidic condition, therefore, the release behaviour of Rod B was investigated in physiological (PBS pH = 7.4) and acidic (5.5) environments (Figure 15a). The cleavage of -C=N- bond leads to separation between dextran and PASP and the controlled release of Rod B could be achieved. The fast hydrolysis of the Schiff base bond in acidic buffer accelerates the release of Rod B from microcapsule in comparison to release in physiological medium [177]. The same release behaviour of Rod B was achieved by Zhang and co-workers by mixing α-CD attached to dextran and poly(acrylic acid) with photo-switchable *p*-aminoazobenzene end groups. The controlled release has been reached by dissociation of microcapsules after cleavage of IC as a result of shifting the *cis* form of the azo bond to *trans* after UV light irradiation (365 nm). The release studies showed that irradiation dramatically accelerate the release of Rod B from microcapsules within 300 min as shown in Figure 15b,c [178].

Moreover, the bio-based nanoparticles (NPs) composed of naturally occurring polysaccharides can be also used for controllable drug delivery. To prepare reversible, supramolecular systems self-assembled into NPs in water, the alginate modified by β-CD, metoxypolyetylene ferrocene end-capping (Fc-mPEG) and α-CD were mixed. The formation of NPs is based on two supramolecular host-guest interactions: a) creation of IC between β-CD attached to alginates chain and ferrocene end-group of metoxypolyetylene, and b) creation of IC between metoxypolyetylene chain and α-CD. However, the second interaction based on formation IC with α-CD is the determining process of NPs formation. Moreover, this strategy allows for obtaining NPs with a diameter of 100 nm for controlled delivery of the enzyme bovine serum albumin (BSA). The controlled release of BSA from NPs in glucose/glucose oxidase (GOD) environment can be achieved by gentle oxidation of Fc end-group (Figure 14b). The oxidation agent (H_2_O_2_) can be prepared as a by-product of glucose oxidation by GOD. After oxidation of Fc, NPs became unstable as a result of cleavage of the IC between β-CD and further BSA may be released in a controlled manner. Moreover, the presence of β-CD in the NPs structure enhance the encapsulation efficiency [184]. Also, the well-known interaction between β-CD and adamantane [185] was used to prepared self-assembled NPs from two series of dextran polymers a) dextran modified by β-CD and b) dextran modified by adamantyl groups. After simple mixing of these supramolecular polymers, the IC between β-CD and adamantyl end group is formed and the NPs with a diameter ranging from 100 to 250 nm could be obtained. The NPs were formed only when the degree of the β-CD substitution in dextran was higher than 4%, and, as the amount of β-CD increased, the NP size decreased whereas the stability of obtained NPs decreased with increasing degree of β-CD substitution [186].

Chitosan (CS) is a natural cationic polysaccharide used for the preparation drug delivery systems due to its biological activity and ability for effective entrapping of cargo molecules [187]. However, the poor solubility of CS in water or common organic solvent causes low reactivity and limits its application. To overcome this limitation some derivatives of CS for instance, maleoyl chitosan [179] or thiolated chitosan [180] were prepared. The most common reaction allowing the attachment β-CD to CS chain is the reaction with *p*-toluenosulfonyl derivatives [179,181,188,189]. This strategy leads to chitosan drug delivery systems functionalized with β-CD moieties for drug [179], DNA [190] or protein delivery [181]. To prepare hydrogels based on chitosan derivatives, the metoxypolyetylene glycol-*co*-polycaprolactone carboxyl-terminated (mPEG-PCL) was attached to chitosan backbone. It was observed that the amphiphilic nature of prepared copolymer cannot lead to the hydrogel formation in room or slightly elevated temperature. However, after mixing of 2.5% water solution of the copolymer with 12% aqueous solution of α-CD within a few minutes or seconds rapid formation of hydrogel occurred as a result of IC formation between mPEG-PCL chains and α-CD cavity. To estimate the release profile, the BSA loaded hydrogels was also prepared by adding BSA to the α-CD solution. The release rate of BSA from obtained hydrogel was strongly dependent on grafting degree, copolymer and α-CD concentration. The release profiles of BSA from obtained hydrogels are significantly faster as compared with reported before for triblock copolymer [poly(ethylene glycol-*co*-3-hydroxybutyrate-*co*-ethylene glycol)] with the α-CD moiety [147,182]. The preparation of supramolecular hydrogels based on PLA attached to the chitosan chain was also shown by Hu et al. In this work, the ability to the formation of the IC between PLA chain and β-CD was employed to prepared self-assembly hydrogel for simultaneous delivery of BSA and Heparin. Furthermore, the presence of weak, labile interaction makes the polymer sensitive for temperature. Due to the temperature-sensitiveness of prepared hydrogels, the release profile was investigated as a function of temperature. The increase in temperature from 20 °C to 30 °C causes an increase of release rate of about 27% for heparin and 13% for BSA [191]. The CD cavity can be also used for purification of water from micropollutants. Sillanpaa et al. insoluble EDTA-crosslinked chitosan bearing β-CD trifunctional material for water purification. The one-pot synthesis leads for preparation high efficient network for metal and drugs removal [188].

This section introduces the aspects of natural polysaccharides containing cyclodextrin moiety as a drug delivery systems. with This section introduces the aspects of natural polysaccharides containing cyclodextrin moiety as a drug delivery systems. Currently alginates, dextran, chitosan and its derivatives are widely used in the field of biomedical applications due to its beneficial properties. However, the polysaccharides require modification to be used as a drug delivery system. In this respect, the polymers or organic compounds could be successfully used to modify polysaccharides and thus enhance theirs low reactivity and solubility in water or organic solvents. The combination of CDs with different types of polysaccharides provides new hybrid materials which successfully slow down the release and reduce the side effects of the drug. Additionally, the CDs used as a crosslinking agent might enhance the unsatisfactory mechanical properties of bio-based materials. However, still existing limitations give opportunities to search for new solutions to improve the utility of polysaccharides as drug delivery systems.

## 6. Conclusions

In summary, by the construction of complex systems based on biocompatible polymers and CDs, a variety of multifunctional materials were developed. The development in macromolecular synthesis allows for the preparation of supramolecular assemblies with various architectures. Typically, nano- or micro-particle, hydrogels, and fibers are used as drug delivery carriers. It was demonstrated that these extraordinary materials, after efficient drug loading, show the controllable drug release by taking advantage of host-guest interactions and other noncovalent forces. The liberation of several important biologically active agents, including antibiotics, vitamins, hormones, enzymes, anticancer drugs, nonsteroidal anti-inflammatory drugs, and physiologically active lipid compounds has been examined in vitro. Although gradual progress has been achieved in the preparation of systems containing biocompatible polymers and cyclodextrins, some issues remain a challenge. Especially, when the synthetic strategy involves multiple steps procedure, the precise control on the composition and architecture of the final material appears to be difficult to accomplish. Moreover, the problem concerning the toxicity and pharmacokinetic study of these cyclodextrin-based carriers within the body should be investigated because most of the information is based on in vitro cell models. Besides, the comprehensive evaluation of the biodegradability of CD-based supramolecular drug delivery systems is still desired. Nevertheless, the advanced technology which exploits the advantages of cyclodextrin and biocompatible polymers represents a promising and innovative strategy allowing drug carriers design and preparation. Bearing in mind the extraordinary features of the system, further work should be intensified to obtain new biomedical materials for future therapies.

## Figures and Tables

**Table 2:** The summary of described micro- and nanoparticles, fibers, and hydrogels for drug delivery based on a combination of PCL and its copolymers with different CDs.

Type of Drug Delivery System	Platform	Type of CD	Drug/Biomolecule	Release Medium	In Vitro/In Vivo Studies	Ref.
Microparticle	PCL	HP-β-CD	Diffractaic acid	pH 7.4	Vero cells	[61]
Nanoparticle	PCL	α-CD	Camptothecin/DOX	PBS	HepG2 cells	[63]
Hydrogel	PCL-*b*-PEG-*b*-PCL	β-CD	Indomethacin	PBS	Rat paws	[64]
Nanoparticle	PCL-*b*-PEG	β-CD	DOX	PBS	SKOV3 cells/HEK293T cells	[65]
Nanofiber	PCL	HP-β-CD	Sulfisoxazole	PBS	nd	[68]
Hydrogel	PCL-*b*-PEG	α-CD	Diclofenac	PBS	L-929 cells/HCEC cells/rabbit eyes	[69]
Hydrogel	(mPEG acetal- PCL-acetal-)_3_	α-CD	DOX hydrochloride	pH 5.0, pH 7.4	nd	[70]
Nanofiber	PCL	β-CD	α-Tocopherol	PBS	nd	[71]
Nanofiber	PCL	β-CD	Naproxen	PBS	nd	[72]
Nanofiber	PCL	β-CD	Ciprofloxacin	pH 7.2	nd	[73]
Nanofiber	PCL	γ-CD	Oxidoreductase	pH 7.5	Catalase enzyme/	[74]
Nanofiber	PCL	γ-CD	Oxidoreductase	pH 7.5	Laccase enzyme	[75]
Nanofiber	PCL	β-CD	Silver sulfadiazine	PBS	*E. coli/Klebsiella pneumoniae/S. epidermidis/S. aureus*	[76]
Nanofiber	PCL	β-CD	Tetracycline	PBS	*Aggregatibacteractinomycetem comitans/Porphyromonas gingivalis*	[77]
Nanoparticle	PCL-*b*-PEG	HP-β-CD	Docetaxel	PBS	MCF-7 cells	[78]
Nanoparticle	PCL-*b*-PEG	HP-β-CD	Paclitaxel/Cidofovir	pH 5.5	L929 cells,	[79]
Nanoparticle	PCL-*b*-PEG	β-CD	Paclitaxel	PBS	HepG2 cells	[80]
Nanoparticle	methoxy-PEG-*b*-PCL	Triacetyl-β-CD	Sepiapterin	PBS	nd	[81]
Nanoparticle	PCL-*b*-PEG	β-CD	DOX	pH 7.0–2.0	nd	[82]
Nanoparticle	Star-PCL-PEG	DMβ-CD	DOX	PBS/1,4-dithio- threitol solution	Skov cells/HEK293 T cells/ tumor-bearing mouse	[83]
Nanoparticle	Star-PCL-PEG	β-CD	DOX	PBS	A549 cells	[84]
Nanoparticle	Star-PCL-PEG	β-CD	DOX	PBS	HeLa cells	[85]
Hydrogel	PCL-*b*-PEG-*b*-PCL	γ-CD	Insulin	PBS	nd	[86]
Hydrogel	PCL-*b*-PEG-*b*-PCL	α-CD	B_12_ vitamin	PBS	nd	[87]
Hydrogel	PCL-*b*-PEG and poly(ethylene glycol)-*b*-poly(acrylic acid)	α-CD	DOX/Cisplatin	PBS	Human bladder carcinoma EJ cells	[88]
Hydrogel	methoxy PEG)-*b*-PCL-co-1,4,8-trioxa-[4.6]spiro-9-un-decanone	α-CD	Paclitaxel	PBS	HeLa cells/7703 cells	[89]
Hydrogel	mPEG–PCL–mPEG	α-CD	Erythropoietin	PBS	Rats heart muscle	[90]
Hydrogel	mPEG–b-PCL-b-poly[2-(dimethylamino) ethyl methacrylate	α-CD	DNA Polyplexes	PBS	COS7 cells	[91]
Hydrogel	PCL-*b*-PEG-*b*-PCL	γ-CD	Dexamethasone	PBS	nd	[92]
Nanoparticle	PCL-*b*-PEG	HP-β-CD	Docetaxel	PBS	HeLa cells	[93]

**Table 4:** The summary of described hydrogels, microparticles and fibres for drug delivery based on combination of polysaccharides and its copolymers with different CDs.

Type of Drug Delivery System	Platform	Type of CD	Drug	Release Medium	In Vitro Studies	Ref.
Nanoparticle	Alginates	β-CD	BSA	PBS	CT26 cells	[174]
Hydrogel	Alginates	β-CD	Methyl orange	0.9% NaCl	nd	[175]
Hydrogel	Alginates	β-CD	Retinolic acid	PBS	Zebrafish embryos	[176]
Microcapsule	Dextran	α-CD	Rhodamine B	PBS or pH 5.5	HeLa cells	[177]
Microcapsule	Dextran	α-CD	Rhodamine B	PBS	nd	[178]
Nanoparticle	Chitosan	β-CD	Ketoprofen	PBS	A549 cells	[179]
Tablet	Chitosan	β-CD	Ketoprofen	pH 6.0	Mouse mucosa	[180]
Nanoparticle	Chitosan	β-CD	Insuline	PBS	NIH 3T3 cells	[181]
Hydrogel	Chitosan/PCL	α-CD	BSA	PBS	nd	[182]

## References

[1] Yang L., Tan X., Wang Z., Zhang X. (2015). Supramolecular polymers: Historical development, preparation, characterization, and functions. Chem. Rev..

[2] Geng W.C., Sessler J.L., Guo D.S. (2020). Supramolecular prodrugs based on host-guest interactions. Chem. Soc. Rev..

[3] Hu Q.D., Tang G.P., Chu P.K. (2014). Cyclodextrin-based host-guest supramolecular nanoparticles for delivery: From design to applications. Acc. Chem. Res..

[4] Zhou J., Ritter H. (2010). Cyclodextrin functionalized polymers as drug delivery systems. Polym. Chem..

[5] Rusa C.C., Bullions T.A., Fox J., Porbeni F.E., Wang X., Tonelli A.E. (2002). Inclusion compound formation with a new columnar cyclodextrin host. Langmuir.

[6] Connors K.A. (1997). The stability of cyclodextrin complexes in solution. Chem. Rev..

[7] Takashima Y., Osaki M., Harada A. (2004). Cyclodextrin-initiated polymerization of cyclic esters in bulk: Formation of polyester-tethered cyclodextrins. J. Am. Chem. Soc..

[8] Faugeras P.A., Boëns B., Elchinger P.H., Brouillette F., Montplaisir D., Zerrouki R., Lucas R. (2012). When cyclodextrins meet click chemistry. Eur. J. Org. Chem..

[9] Concheiro A., Alvarez-Lorenzo C. (2013). Chemically cross-linked and grafted cyclodextrin hydrogels: From nanostructures to drug-eluting medical devices. Adv. Drug Deliv. Rev..

[10] Yang J.S., Yang L. (2013). Preparation and application of cyclodextrin immobilized polysaccharides. J. Mater. Chem. B.

[11] Wang L., Li L.L., Fan Y.S., Wang H. (2013). Host-guest supramolecular nanosystems for cancer diagnostics and therapeutics. Adv. Mater..

[12] Hettiarachchi G., Nguyen D., Wu J., Lucas D., Ma D., Isaacs L., Briken V. (2010). Toxicology and drug delivery by cucurbit[n]uril type molecular containers. PLoS ONE.

[13] Yan X., Wang F., Zheng B., Huang F. (2012). Stimuli-responsive supramolecular polymeric materials. Chem. Soc. Rev..

[14] Cova T.F., Murtinho D., Pais A.A.C.C., Valente A.J.M. (2018). Combining cellulose and cyclodextrins: Fascinating designs for materials and pharmaceutics. Front. Chem..

[15] Pawar R.P., Tekale S.U., Shisodia S.U., Totre J.T., Domb A.J. (2014). Biomedical applications of poly(lactic acid). Rec. Pat. Regen. Med..

[16] Cheng Y., Deng S., Chen P., Ruan R. (2009). Polylactic acid (PLA) synthesis and modifications: A review. Front. Chem. China.

[17] Xiao R.Z., Zeng Z.W., Zhou G.L., Wang J.J., Li F.Z., Wang A.M. (2010). Recent advances in PEG-PLA block copolymer nanoparticles. Int. J. Nanomed..

[18] Basu A., Kunduru K.R., Doppalapudi S., Domb A.J., Khan W. (2016). Poly(lactic acid) based hydrogels. Adv. Drug Deliv. Rev..

[19] Khodaverdi E., Tekie F.S.M., Hadizadeh F., Esmaeel H., Mohajeri S.A., Tabassi S.A.S., Zohuri G. (2014). Hydrogels composed of cyclodextrin inclusion complexes with PLGA-PEG-PLGA triblock copolymers as drug delivery systems. AAPS PharmSciTech.

[20] Poudel A.J., He F., Huang L., Xiao L., Yang G. (2018). Supramolecular hydrogels based on poly (ethylene glycol)-poly (lactic acid) block copolymer micelles and α-cyclodextrin for potential injectable drug delivery system. Carbohydr. Polym..

[21] Aytac Z., Kusku S.I., Durgun E., Uyar T. (2016). Encapsulation of gallic acid/cyclodextrin inclusion complex in electrospun polylactic acid nanofibers: Release behavior and antioxidant activity of gallic acid. Mater. Sci. Eng. C.

[22] Kost B., Svyntkivska M., Brzeziński M., Makowski T., Piorkowska E., Rajkowska K., Kunicka-Styczyńska A., Biela T. (2020). PLA/β-CD-based fibres loaded with quercetin as potential antibacterial dressing materials. Colloids Surf. B.

[23] Vermet G., Degoutin S., Chai F., Maton M., Flores C., Neut C., Danjou P.E., Martel B., Blanchemain N. (2017). Cyclodextrin modified PLLA parietal reinforcement implant with prolonged antibacterial activity. Acta Biomater..

[24] Bucatariu S., Constantin M., Ascenzi P., Fundueanu G. (2016). Poly(lactide-co-glycolide)/cyclodextrin (polyethyleneimine) microspheres for controlled delivery of dexamethasone. React. Funct. Polym..

[25] Cannavà C., Tommasini S., Stancanelli R., Cardile V., Cilurzo F., Giannone I., Puglisi G., Ventura C.A. (2013). Celecoxib-loaded PLGA/cyclodextrin microspheres: Characterization and evaluation of anti-inflammatory activity on human chondrocyte cultures. Colloids Surf. B.

[26] Gupta V., Davis M., Hope-Weeks L.J., Ahsan F. (2011). PLGA microparticles encapsulating prostaglandin E 1- hydroxypropyl-β-cyclodextrin (PGE 1-HPβCD) complex for the treatment of pulmonary arterial hypertension (PAH). Pharm. Res..

[27] Li F., Wen Y., Zhang Y., Zheng K., Ban J., Xie Q., Wen Y., Liu Q., Chen F., Mo Z. (2019). Characterisation of 2-HP-β-cyclodextrin-PLGA nanoparticle complexes for potential use as ocular drug delivery vehicles. Artif. Cells, Nanomed. Biotechnol..

[28] Li S., He Q., Chen T., Wu W., Lang K., Li Z.M., Li J. (2014). Controlled co-delivery nanocarriers based on mixed micelles formed from cyclodextrin-conjugated and cross-linked copolymers. Colloids Surf. B Biointerfaces.

[29] Zhang Z., Lv Q., Gao X., Chen L., Cao Y., Yu S., He C., Chen X. (2015). pH-Responsive poly(ethylene glycol)/poly(L-lactide) supramolecular micelles based on host-guest interaction. ACS Appl. Mater. Interfaces.

[30] Aytac Z., Keskin N.O.S., Tekinay T., Uyar T. (2017). Antioxidant α-tocopherol/γ-cyclodextrin–inclusion complex encapsulated poly(lactic acid) electrospun nanofibrous web for food packaging. J. Appl. Polym. Sci..

[31] Kayaci F., Umu O.C.O., Tekinay T., Uyar T. (2013). Antibacterial electrospun poly(lactic acid) (PLA) nanofibrous webs incorporating triclosan/cyclodextrin inclusion complexes. J. Agric. Food Chem..

[32] Sudakaran S.V., Venugopal J.R., Vijayakumar G.P., Abisegapriyan S., Grace A.N., Ramakrishna S. (2017). Sequel of MgO nanoparticles in PLACL nanofibers for anti-cancer therapy in synergy with curcumin/β-cyclodextrin. Mater. Sci. Eng. C.

[33] Nilkumhang S., Basit A.W. (2009). The robustness and flexibility of an emulsion solvent evaporation method to prepare pH-responsive microparticles. Int. J. Pharm..

[34] Giri T.K., Choudhary C., Ajazuddin, Alexander A., Badwaik H., Tripathi D.K. (2013). Prospects of pharmaceuticals and biopharmaceuticals loaded microparticles prepared by double emulsion technique for controlled delivery. Saudi Pharm. J..

[35] Malaekeh-nikouei B., Sajadi S.A., Jaafari M.R. (2005). Preparation and characterization of PLGA microspheres loaded by cyclosporine-cyclodextrin complex. Iran. J. Pharm. Sci..

[36] Brzeziński M., Kost B., Wedepohl S., Socka M., Biela T., Calderón M. (2019). Stereocomplexed PLA microspheres: Control over morphology, drug encapsulation and anticancer activity. Colloids Surf. B.

[37] Uekama K., Hieda Y., Hirayama F., Arima H., Sudoh M., Yagi A., Terashima H. (2001). Stabilizing and solubilizing effects of sulfobutyl ether β-cyclodextrin on prostaglandin E1 analogue. Pharm. Res..

[38] Çirpanli Y., Bilensoy E., Lale Doǧan A., Çaliş S. (2009). Comparative evaluation of polymeric and amphiphilic cyclodextrin nanoparticles for effective camptothecin delivery. Eur. J. Pharm. Biopharm..

[39] He Q., Wu W., Xiu K., Zhang Q., Xu F., Li J. (2013). Controlled drug release system based on cyclodextrin-conjugated poly(lactic acid)-b-poly(ethylene glycol) micelles. Int. J. Pharm..

[40] Gu W.X., Zhu M., Song N., Du X., Yang Y.W., Gao H. (2015). Reverse micelles based on biocompatible β-cyclodextrin conjugated polyethylene glycol block polylactide for protein delivery. J. Mater. Chem. B.

[41] Zhang L., Lu J., Jin Y., Qiu L. (2014). Folate-conjugated beta-cyclodextrin-based polymeric micelles with enhanced doxorubicin antitumor efficacy. Colloids Surf. B.

[42] Kost B., Brzeziński M., Cieślak M., Królewska-Golińska K., Makowski T., Socka M., Biela T. (2019). Stereocomplexed micelles based on polylactides with β-cyclodextrin core as anti-cancer drug carriers. Eur. Polym. J..

[43] Gao H., Wang Y.N., Fan Y.G., Ma J.B. (2005). Synthesis of a biodegradable tadpole-shaped polymer via the coupling reaction of polylactide onto mono(6-(2-aminoethyl)amino-6-deoxy)-β- cyclodextrin and its properties as the new carrier of protein delivery system. J. Control. Release.

[44] Gao H., Yang Y.W., Fan Y.G., Ma J.B. (2006). Conjugates of poly(dl-lactic acid) with ethylenediamino or diethylenetriamino bridged bis(β-cyclodextrin)s and their nanoparticles as protein delivery systems. J. Control. Release.

[45] Gao H., Wang Y.-N., Fan Y.-G., Ma J.-B. (2007). Conjugates of poly(DL-lactide-co-glycolide) on amino cyclodextrins and their nanoparticles as protein delivery system. J. Biomed. Mater. Res. Part A.

[46] Li W., Fan X., Wang X., Shang X., Wang Q., Lin J., Hu Z., Li Z. (2018). Stereocomplexed micelle formation through enantiomeric PLA-based Y-shaped copolymer for targeted drug delivery. Mater. Sci. Eng. C.

[47] Jiang Q., Zhang Y., Zhuo R., Jiang X. (2016). Supramolecular host-guest polycationic gene delivery system based on poly(cyclodextrin) and azobenzene-terminated polycations. Colloids Surf. B.

[48] Feng X., Ding J., Gref R., Chen X. (2017). Poly(β-cyclodextrin)-mediated polylactide-cholesterol stereocomplex micelles for controlled drug delivery. Chin. J. Polym. Sci..

[49] Yi W.J., Li L.J., He H., Hao Z., Liu B., Chao Z.S., Shen Y. (2018). Synthesis of poly(l-lactide)/β-cyclodextrin/citrate network modified hydroxyapatite and its biomedical properties. New J. Chem..

[50] Woodruff M.A., Hutmacher D.W. (2010). The return of a forgotten polymer-Polycaprolactone in the 21st century. Prog. Polym. Sci..

[51] Labet M., Thielemans W. (2009). Synthesis of polycaprolactone: A review. Chem. Soc. Rev..

[52] Sinha V.R., Bansal K., Kaushik R., Kumria R., Trehan A. (2004). Poly-ϵ-caprolactone microspheres and nanospheres: An overview. Int. J. Pharm..

[53] Dash T.K., Konkimalla V.B. (2012). Poly-є-caprolactone based formulations for drug delivery and tissue engineering: A review. J. Control. Release.

[54] Lecomte P., Riva R., Schmeits S., Rieger J., Van Butsele K., Jérôme C., Jérôme R. (2006). New prospects for the grafting of functional groups onto aliphatic polyesters. ring-opening polymerization of α- or γ-substitutedε-caprolactone followed by chemical derivatization of the substituents. Macromol. Symp..

[55] Seyednejad H., Ghassemi A.H., van Nostrum C.F., Vermonden T., Hennink W.E. (2011). Functional aliphatic polyesters for biomedical and pharmaceutical applications. J. Control. Release.

[56] Parrish B., Breitenkamp R.B., Emrick T. (2005). PEG- and peptide-grafted aliphatic polyesters by click chemistry. J. Am. Chem. Soc..

[57] Hu Y., Zhang L., Cao Y., Ge H., Jiang X., Yang C. (2004). Degradation behavior of poly(ε-caprolactone)-b-poly(ethylene glycol)-b-poly(ε-caprolactone) micelles in aqueous solution. Biomacromolecules.

[58] Dorj B., Kim M.-K., Won J.-E., Kim H.-W. (2011). Functionalization of poly(caprolactone) scaffolds by the surface mineralization for use in bone regeneration. Mater. Lett..

[59] Sousa I., Mendes A., Pereira R.F., Bártolo P.J. (2014). Collagen surface modified poly(ε-caprolactone) scaffolds with improved hydrophilicity and cell adhesion properties. Mater. Lett..

[60] Kawaguchi Y., Nishiyama T., Okada M., Kamachi M., Harada A. (2000). Complex formation of poly(ε-caprolactone) with cyclodextrins. Macromolecules.

[61] Silva C.V.N.S., Barbosa J.A.P., Ferraz M.S., Silva N.H., Honda N.K., Rabello M.M., Hernandes M.Z., Bezerra B.P., Cavalcanti I.M.F., Ayala A.P. (2016). Molecular modeling and cytotoxicity of diffractaic acid: HP-β-CD inclusion complex encapsulated in microspheres. Int. J. Biol. Macromol..

[62] Puglisi A., Bayir E., Timur S., Yagci Y. (2019). pH-Responsive polymersome microparticles as smart cyclodextrin-releasing agents. Biomacromolecules.

[63] Li Y., Chen Y., Dong H., Dong C. (2015). Supramolecular, prodrug-based micelles with enzyme-regulated release behavior for controlled drug delivery. Med. Chem. Comm..

[64] Wei X., Lv X., Zhao Q., Qiu L. (2013). Thermosensitive β-cyclodextrin modified poly(ε-caprolactone)- poly(ethylene glycol)-poly(ε-caprolactone) micelles prolong the anti-inflammatory effect of indomethacin following local injection. Acta Biomater..

[65] Li J., Li X., Liu H., Ren T., Huang L., Deng Z., Yang Y., Zhong S. (2019). GSH and light dual stimuli-responsive supramolecular polymer drug carriers for cancer therapy. Polym. Degrad. Stab..

[66] Xu S., Yin L., Xiang Y., Deng H., Deng L., Fan H., Tang H., Zhang J., Dong A. (2016). Supramolecular hydrogel from nanoparticles and cyclodextrins for local and sustained nanoparticle delivery. Macromol. Biosci..

[67] Narayanan G., Shen J., Boy R., Gupta B.S., Tonelli A.E. (2018). Aliphatic polyester nanofibers functionalized with cyclodextrins and cyclodextrin-guest inclusion complexes. Polymers.

[68] Aytac Z., Sen H.S., Durgun E., Uyar T. (2015). Sulfisoxazole/cyclodextrin inclusion complex incorporated in electrospun hydroxypropyl cellulose nanofibers as drug delivery system. Colloids Surf. B.

[69] Zhang Z., He Z., Liang R., Ma Y., Huang W., Jiang R., Shi S., Chen H., Li X. (2016). Fabrication of a micellar supramolecular hydrogel for ocular drug delivery. Biomacromolecules.

[70] Hu J., Zhang M., He J., Ni P. (2016). Injectable hydrogels by inclusion complexation between a three-armed star copolymer (mPEG-acetal-PCL-acetal-)3 and α-cyclodextrin for pH-triggered drug delivery. RSC Adv..

[71] Aytac Z., Uyar T. (2016). Antioxidant activity and photostability of α-tocopherol/β-cyclodextrin inclusion complex encapsulated electrospun polycaprolactone nanofibers. Eur. Polym. J..

[72] Canbolat M.F., Celebioglu A., Uyar T. (2014). Drug delivery system based on cyclodextrin-naproxen inclusion complex incorporated in electrospun polycaprolactone nanofibers. Colloids Surf. B.

[73] Masoumi S., Amiri S., Bahrami S.H. (2018). PCL-based nanofibers loaded with ciprofloxacin/cyclodextrin containers. J. Text. Inst..

[74] Canbolat M.F., Savas H.B., Gultekin F. (2017). Improved catalytic activity by catalase immobilization using γ-cyclodextrin and electrospun PCL nanofibers. J. Appl. Polym. Sci..

[75] Canbolat M.F., Savas H.B., Gultekin F. (2017). Enzymatic behavior of laccase following interaction with γ-CD and immobilization into PCL nanofibers. Anal. Biochem..

[76] Souza S.O.L., Cotrim M.A.P., Oréfice R.L., Carvalho S.G., Dutra J.A.P., de Paula Careta F., Resende J.A., Villanova J.C.O. (2018). Electrospun poly(ε-caprolactone) matrices containing silver sulfadiazine complexed with β-cyclodextrin as a new pharmaceutical dosage form to wound healing: Preliminary physicochemical and biological evaluation. J. Mater. Sci. Mater. Med..

[77] Monteiro A.P.F., Rocha C.M.S.L., Oliveira M.F., Gontijo S.M.L., Agudelo R.R., Sinisterra R.D., Cortés M.E. (2017). Nanofibers containing tetracycline/β-cyclodextrin: Physico-chemical characterization and antimicrobial evaluation. Carbohydr. Polym..

[78] Varan C., Bilensoy E. (2014). Development of implantable hydroxypropyl-β-cyclodextrin coated polycaprolactone nanoparticles for the controlled delivery of docetaxel to solid tumors. J. Incl. Phenom. Macrocycl. Chem..

[79] Varan C., Wickström H., Sandler N., Aktaş Y., Bilensoy E. (2017). Inkjet printing of antiviral PCL nanoparticles and anticancer cyclodextrin inclusion complexes on bioadhesive film for cervical administration. Int. J. Pharm..

[80] Ahmed A., Wang H., Yu H., Zhou Z.Y., Ding Y., Hu Y. (2015). Surface engineered cyclodextrin embedded polymeric nanoparticles through host-guest interaction used for drug delivery. Chem. Eng. Sci..

[81] Kuplennik N., Sosnik A. (2019). Enhanced nanoencapsulation of sepiapterin within PEG-PCL nanoparticles by complexation with triacetyl-beta cyclodextrin. Molecules.

[82] Gao Y., Li G., Zhou Z., Guo L., Liu X. (2017). Supramolecular assembly of poly(β-cyclodextrin) block copolymer and benzimidazole-poly(ε-caprolactone) based on host-guest recognition for drug delivery. Colloids Surf. B.

[83] Li X., Liu H., Li J., Deng Z., Li L., Liu J., Yuan J., Gao P., Yang Y., Zhong S. (2019). Micelles via self-assembly of amphiphilic beta-cyclodextrin block copolymers as drug carrier for cancer therapy. Colloids Surf. B.

[84] Peng L., Wang Z., Feng A., Huo M., Fang T., Wang K., Wei Y., Yuan J. (2016). Star amphiphilic supramolecular copolymer based on host-guest interaction for electrochemical controlled drug delivery. Polymer.

[85] Zuo C., Peng J., Cong Y., Dai X., Zhang X., Zhao S., Zhang X., Ma L., Wang B., Wei H. (2018). Fabrication of supramolecular star-shaped amphiphilic copolymers for ROS-triggered drug release. J. Colloid Interface Sci..

[86] Khodaverdi E., Heidari Z., Tabassi S.A.S., Tafaghodi M., Alibolandi M., Tekie F.S.M., Khameneh B., Hadizadeh F. (2014). Injectable supramolecular hydrogel from insulin-loaded triblock PCL-PEG-PCL copolymer and γ-cyclodextrin with sustained-release property. AAPS PharmSciTech.

[87] Tabassi S.A.S., Tekie F.S.M., Hadizadeh F., Rashid R., Khodaverdi E., Mohajeri S.A. (2014). Sustained release drug delivery using supramolecular hydrogels of the triblock copolymer PCL-PEG-PCL and α-cyclodextrin. J. Sol-Gel Sci. Technol..

[88] Zhu W., Li Y., Liu L., Chen Y., Xi F. (2012). Supramolecular hydrogels as a universal scaffold for stepwise delivering Dox and Dox/cisplatin loaded block copolymer micelles. Int. J. Pharm..

[89] Yin L., Xu S., Feng Z., Deng H., Zhang J., Gao H., Deng L., Tang H., Dong A. (2017). Supramolecular hydrogel based on high-solid-content mPECT nanoparticles and cyclodextrins for local and sustained drug delivery. Biomater. Sci..

[90] Wang T., Jiang X.-J., Lin T., Ren S., Li X.-Y., Zhang X.-Z., Tang Q. (2009). The inhibition of postinfarct ventricle remodeling without polycythaemia following local sustained intramyocardial delivery of erythropoietin within a supramolecular hydrogel. Biomaterials.

[91] Li Z., Yin H., Zhang Z., Li Liu K., Li J. (2012). Supramolecular anchoring of DNA polyplexes in cyclodextrin-based polypseudorotaxane hydrogels for sustained gene delivery. Biomacromolecules.

[92] Khodaverdi E., Gharechahi M., Alibolandi M., Tekie F.M., Khashyarmanesh B., Hadizadeh F. (2016). Self-assembled supramolecular hydrogel based on PCL-PEG-PCL triblock copolymer and γ-cyclodextrin inclusion complex for sustained delivery of dexamethasone. Int. J. Pharm. Investig..

[93] Conte C., Ungaro F., Maglio G., Tirino P., Siracusano G., Sciortino M.T., Leone N., Palma G., Barbieri A., Arra C. (2013). Biodegradable core-shell nanoassemblies for the delivery of docetaxel and Zn(II)-phthalocyanine inspired by combination therapy for cancer. J. Control. Release.

[94] Park J., Ye M., Park K. (2005). Biodegradable polymers for microencapsulation of drugs. Molecules.

[95] O’Donnell P.B., McGinity J.W. (1997). Preparation of microspheres by the solvent evaporation technique. Adv. Drug Deliv. Rev..

[96] MacEwan S.R., Chilkoti A. (2017). From composition to cure: A systems engineering approach to anticancer drug carriers. Angew. Chemie Int. Ed..

[97] Simões S.M.N., Rey-Rico A., Concheiro A., Alvarez-Lorenzo C. (2015). Supramolecular cyclodextrin-based drug nanocarriers. Chem. Commun..

[98] Oster M., Schlatter G., Gallet S., Baati R., Pollet E., Gaillard C., Avérous L., Fajolles C., Hébraud A. (2017). The study of the pseudo-polyrotaxane architecture as a route for mild surface functionalization by click chemistry of poly(ε-caprolactone)-based electrospun fibers. J. Mater. Chem. B.

[99] Narayanan G., Gupta B.S., Tonelli A.E. (2014). Poly(ε-caprolactone) Nanowebs Functionalized with α- and γ-Cyclodextrins. Biomacromolecules.

[100] Narayanan G., Ormond B.R., Gupta B.S., Tonelli A.E. (2015). Efficient wound odor removal by β-cyclodextrin functionalized poly (ε-caprolactone) nanofibers. J. Appl. Polym. Sci..

[101] Narayanan G., Aguda R., Hartman M., Chung C.-C., Boy R., Gupta B.S., Tonelli A.E. (2015). Fabrication and characterization of poly(ε-caprolactone)/α-cyclodextrin pseudorotaxane nanofibers. Biomacromolecules.

[102] Zhan J., Singh A., Zhang Z., Huang L., Elisseeff J.H. (2012). Multifunctional aliphatic polyester nanofibers for tissue engineering. Biomatter.

[103] Panday R., Poudel A.J., Li X., Adhikari M., Ullah M.W., Yang G. (2018). Amphiphilic core-shell nanoparticles: Synthesis, biophysical properties, and applications. Colloids Surf. B.

[104] Boarca B., Lungu I.I., Holban A.M. (2019). Core–shell nanomaterials for infection and cancer therapy. Materials for Biomedical Engineering.

[105] Chen G., Wang Y., Xie R., Gong S. (2018). A review on core–shell structured unimolecular nanoparticles for biomedical applications. Adv. Drug Deliv. Rev..

[106] Zhao J., Weng G., Li J., Zhu J., Zhao J. (2018). Polyester-based nanoparticles for nucleic acid delivery. Mater. Sci. Eng. C.

[107] Yi Y., Lin G., Chen S., Liu J., Zhang H., Mi P. (2018). Polyester micelles for drug delivery and cancer theranostics: Current achievements, progresses and future perspectives. Mater. Sci. Eng. C.

[108] Alexander A., Dwivedi S., Ajazuddin, Giri T.K., Saraf S., Saraf S., Tripathi D.K. (2012). Approaches for breaking the barriers of drug permeation through transdermal drug delivery. J. Control. Release.

[109] Conte C., Costabile G., d’Angelo I., Pannico M., Musto P., Grassia G., Ialenti A., Tirino P., Miro A., Ungaro F. (2015). Skin transport of PEGylated poly(ε-caprolactone) nanoparticles assisted by (2-hydroxypropyl)-β-cyclodextrin. J. Colloid Interface Sci..

[110] Lu Y., Zou H., Yuan H., Gu S., Yuan W., Li M. (2017). Triple stimuli-responsive supramolecular assemblies based on host-guest inclusion complexation between β-cyclodextrin and azobenzene. Eur. Polym. J..

[111] Liu X., Chen B., Li X., Zhang L., Xu Y., Liu Z., Cheng Z., Zhu X. (2015). Self-assembly of BODIPY based pH-sensitive near-infrared polymeric micelles for drug controlled delivery and fluorescence imaging applications. Nanoscale.

[112] Cameron D.J.A., Shaver M.P. (2011). Aliphatic polyester polymer stars: Synthesis, properties and applications in biomedicine and nanotechnology. Chem. Soc. Rev..

[113] Yin H., Kang S.-W., Han Bae Y. (2009). Polymersome formation from AB2 type 3-miktoarm star copolymers. Macromolecules.

[114] Wang F., Bronich T.K., Kabanov A.V., David Rauh R., Roovers J. (2008). Synthesis and characterization of star poly(ε-caprolactone)-b-poly(ethylene glycol) and poly(l-lactide)-b-poly(ethylene glycol) copolymers: Evaluation as drug delivery carriers. Bioconjug. Chem..

[115] Gou P.-F., Zhu W.-P., Xu N., Shen Z.-Q. (2010). Synthesis and self-assembly of well-defined cyclodextrin-centered amphiphilic A14B7 multimiktoarm star copolymers based on poly(ε-caprolactone) and poly(acrylic acid). J. Polym. Sci. Part A Polym. Chem..

[116] Gou P.-F., Zhu W.-P., Shen Z.-Q. (2010). Synthesis, self-assembly, and drug-loading capacity of well-defined cyclodextrin-centered drug-conjugated amphiphilic A14B7 miktoarm star copolymers based on poly(ε-caprolactone) and poly(ethylene glycol). Biomacromolecules.

[117] Yasen W., Dong R., Zhou L., Wu J., Cao C., Aini A., Zhu X. (2017). Synthesis of a cationic supramolecular block copolymer with covalent and noncovalent polymer blocks for gene delivery. ACS Appl. Mater. Interfaces.

[118] Fu C., Lin X., Wang J., Zheng X., Li X., Lin Z., Lin G. (2016). Injectable micellar supramolecular hydrogel for delivery of hydrophobic anticancer drugs. J. Mater. Sci. Mater. Med..

[119] Wu D.-Q., Wang T., Lu B., Xu X.-D., Cheng S.-X., Jiang X.-J., Zhang X.-Z., Zhuo R.-X. (2008). Fabrication of supramolecular hydrogels for drug delivery and stem cell encapsulation. Langmuir.

[120] Veronese F.M., Mero A. (2008). The impact of PEGylation on biological therapies. BioDrugs.

[121] Milton Harris J., Chess R.B. (2003). Effect of pegylation on pharmaceuticals. Nat. Rev. Drug Discov..

[122] Joralemon M.J., McRae S., Emrick T. (2010). PEGylated polymers for medicine: From conjugation to self-assembled systems. Chem. Commun..

[123] Rojas-Aguirre Y., Torres-Mena M.A., López-Méndez L.J., Alcaraz-Estrada S.L., Guadarrama P., Urucha-Ortíz J.M. (2019). PEGylated β-cyclodextrins: Click synthesis and in vitro biological insights. Carbohydr. Polym..

[124] Liang L., Astruc D. (2011). The copper(I)-catalyzed alkyne-azide cycloaddition (CuAAC) “click” reaction and its applications. An overview. Coord. Chem. Rev..

[125] Gaetke L.M., Chow-Johnson H.S., Chow C.K. (2014). Copper: Toxicological relevance and mechanisms. Arch. Toxicol..

[126] Hashidzume A., Tomatsu I., Harada A. (2006). Interaction of cyclodextrins with side chains of water soluble polymers: A simple model for biological molecular recognition and its utilization for stimuli-responsive systems. Polymer.

[127] Antoniuk I., Plazzotta B., Wintgens V., Volet G., Nielsen T.T., Pedersen J.S., Amiel C. (2017). Host–guest interaction and structural ordering in polymeric nanoassemblies: Influence of molecular design. Int. J. Pharm..

[128] Huang F., Gibson H.W. (2005). Polypseudorotaxanes and polyrotaxanes. Prog. Polym. Sci..

[129] Shinohara K., Yamashita M., Uchida W., Okabe C., Oshima S., Sugino M., Egawa Y., Miki R., Hosoya O., Fujihara T. (2014). Preparation of polypseudorotaxanes composed of cyclodextrin and polymers in microspheres. Chem. Pharm. Bull..

[130] Moon C., Kwon Y.M., Lee W.K., Park Y.J., Yang V.C. (2007). In vitro assessment of a novel polyrotaxane-based drug delivery system integrated with a cell-penetrating peptide. J. Control. Release.

[131] Xu T., Li J., Cao J., Gao W., Li L., He B. (2017). The effect of α-cyclodextrin on poly(pseudo)rotaxane nanoparticles self-assembled by protoporphyrin modified poly(ethylene glycol) for anticancer drug delivery. Carbohydr. Polym..

[132] Yang C., Qin Y., Tu K., Xu C., Li Z., Zhang Z. (2018). Star-shaped polymer of β-cyclodextrin-g-vitamin E TPGS for doxorubicin delivery and multidrug resistance inhibition. Colloids Surf. B.

[133] Fan W., Xu Y., Li Z., Li Q. (2019). Folic acid-modified β-cyclodextrin nanoparticles as drug delivery to load DOX for liver cancer therapeutics. Soft Mater..

[134] Hyun H., Lee S., Lim W., Jo D., Jung J.S., Jo G., Kim S.Y., Lee D.-W., Um S., Yang D.H. (2019). Engineered beta-cyclodextrin-based carrier for targeted doxorubicin delivery in breast cancer therapy in vivo. J. Ind. Eng. Chem..

[135] Wang Y., Wang H., Chen Y., Liu X., Jin Q., Ji J. (2014). PH and hydrogen peroxide dual responsive supramolecular prodrug system for controlled release of bioactive molecules. Colloids Surf. B.

[136] Chen X., Yao X., Wang C., Chen L., Chen X. (2015). Hyperbranched PEG-based supramolecular nanoparticles for acid-responsive targeted drug delivery. Biomater. Sci..

[137] Xiong Q., Cui M., Yu G., Wang J., Song T. (2018). Facile fabrication of reduction-responsive supramolecular nanoassemblies for co-delivery of doxorubicin and sorafenib toward hepatoma cells. Front. Pharmacol..

[138] Gérard Yaméogo J.B., Mazet R., Wouessidjewe D., Choisnard L., Godin-Ribuot D., Putaux J.L., Semdé R., Gèze A. (2020). Pharmacokinetic study of intravenously administered artemisinin-loaded surface-decorated amphiphilic γ-cyclodextrin nanoparticles. Mater. Sci. Eng. C.

[139] Feng R., Deng P., Zhou F., Feng S., Song Z. (2018). Pluronic F127-cyclodextrin conjugate micelles for encapsulation of honokiol. J. Nanoparticle Res..

[140] Liao R., Yi S., Liu M., Jin W., Yang B. (2015). Folic-acid-targeted self-assembling supramolecular carrier for gene delivery. ChemBioChem.

[141] Mohammed A.F.A., Higashi T., Motoyama K., Ohyama A., Onodera R., Khaled K.A., Sarhan H.A., Hussein A.K., Arima H. (2019). In vitro and in vivo co-delivery of siRNA and doxorubicin by folate-PEG-appended dendrimer/glucuronylglucosyl-β-cyclodextrin conjugate. AAPS J..

[142] Yu Y., Chen C.K., Law W.C., Weinheimer E., Sengupta S., Prasad P.N., Cheng C. (2014). Polylactide-graft-doxorubicin nanoparticles with precisely controlled drug loading for pH-triggered drug delivery. Biomacromolecules.

[143] Tran T.V., Vo U.V., Pham D.Y., Tran D.L., Nguyen T.H., Tran N.Q., Nguyen C.K., Thu L.V., Nguyen D.H. (2016). Supramolecular chemistry at interfaces: Host-guest interactions for attaching PEG and 5-fluorouracil to the surface of porous nanosilica. Green Process. Synth..

[144] Nguyen Thi T.T., Tran T.V., Tran N.Q., Nguyen C.K., Nguyen D.H. (2017). Hierarchical self-assembly of heparin-PEG end-capped porous silica as a redox sensitive nanocarrier for doxorubicin delivery. Mater. Sci. Eng. C.

[145] Sawant V.J., Bamane S.R. (2018). PEG-beta-cyclodextrin functionalized zinc oxide nanoparticles show cell imaging with high drug payload and sustained pH responsive delivery of curcumin in to MCF-7 cells. J. Drug Deliv. Sci. Technol..

[146] Klaewklod A., Tantishaiyakul V., Hirun N., Sangfai T., Li L. (2015). Characterization of supramolecular gels based on β-cyclodextrin and polyethyleneglycol and their potential use for topical drug delivery. Mater. Sci. Eng. C.

[147] Li J., Li X., Ni X., Wang X., Li H., Leong K.W. (2006). Self-assembled supramolecular hydrogels formed by biodegradable PEO-PHB-PEO triblock copolymers and α-cyclodextrin for controlled drug delivery. Biomaterials.

[148] Salmaso S., Semenzato A., Bersani S., Matricardi P., Rossi F., Caliceti P. (2007). Cyclodextrin/PEG based hydrogels for multi-drug delivery. Int. J. Pharm..

[149] Wang J., Williamson G.S., Yang H. (2018). Branched polyrotaxane hydrogels consisting of alpha-cyclodextrin and low-molecular-weight four-arm polyethylene glycol and the utility of their thixotropic property for controlled drug release. Colloids Surf. B.

[150] Kuang H., He H., Zhang Z., Qi Y., Xie Z., Jing X., Huang Y. (2014). Injectable and biodegradable supramolecular hydrogels formed by nucleobase-terminated poly(ethylene oxide)s and α-cyclodextrin. J. Mater. Chem. B.

[151] Yamaguchi S., Higashi K., Azuma T., Okamoto A. (2019). Supramolecular polymeric hydrogels for ultrasound-guided protein release. Biotechnol. J..

[152] Khan S., Minhas M.U., Ahmad M., Sohail M. (2018). Self-assembled supramolecular thermoreversible β-cyclodextrin/ethylene glycol injectable hydrogels with difunctional Pluronic^®^127 as controlled delivery depot of curcumin. Development, characterization and in vitro evaluation. J. Biomater. Sci. Polym. Ed..

[153] Yu J., Ha W., Chen J., Shi Y.P. (2014). PH-Responsive supramolecular hydrogels for codelivery of hydrophobic and hydrophilic anticancer drugs. RSC Adv..

[154] Müller C., Schubiger P.A., Schibli R. (2006). In vitro and in vivo targeting of different folate receptor-positive cancer cell lines with a novel 99mTc-radiofolate tracer. Eur. J. Nucl. Med. Mol. Imaging.

[155] Bao Y., Yin M., Hu X., Zhuang X., Sun Y., Guo Y., Tan S., Zhang Z. (2016). A safe, simple and efficient doxorubicin prodrug hybrid micelle for overcoming tumor multidrug resistance and targeting delivery. J. Control. Release.

[156] Zhu D., Tao W., Zhang H., Liu G., Wang T., Zhang L., Zeng X., Mei L. (2016). Docetaxel (DTX)-loaded polydopamine-modified TPGS-PLA nanoparticles as a targeted drug delivery system for the treatment of liver cancer. Acta Biomater..

[157] Brzeziński M., Wedepohl S., Kost B., Calderón M. (2018). Nanoparticles from supramolecular polylactides overcome drug resistance of cancer cells. Eur. Polym. J..

[158] Caló E., Khutoryanskiy V.V. (2015). Biomedical applications of hydrogels: A review of patents and commercial products. Eur. Polym. J..

[159] Wang W., Zhang Y., Liu W. (2017). Bioinspired fabrication of high strength hydrogels from non-covalent interactions. Prog. Polym. Sci..

[160] Klaewklod A., Tantishaiyakul V., Sangfai T., Hirun N., Rugmai S. (2014). Chemometric and experimental investigations of organogelation based on β-cyclodextrin. Adv. Mater. Res..

[161] Van de Manakker F., Van der Pot M., Vermonden T., Van Nostrum C.F., Hennink W.E. (2008). Self-assembling hydrogels based on β-cyclodextrin/cholesterol inclusion complexes. Macromolecules.

[162] Van Manakker F.D., Braeckmans K., Morabit N.E., De Smedt S.C., Van Nostrum C.F., Hennink W.E. (2009). Protein-release behavior of self-assembled PEG-ß-cyclodextrin/PEG- cholesterol hydrogels. Adv. Funct. Mater..

[163] Eliasof S., Lazarus D., Peters C.G., Case R.I., Cole R.O., Hwang J., Schluep T., Chao J., Lin J., Yen Y. (2013). Correlating preclinical animal studies and human clinical trials of a multifunctional, polymeric nanoparticle. Proc. Natl. Acad. Sci. USA.

[164] Ren Y., Bai Y., Zhang Z., Cai W., Del Rio Flores A. (2019). The preparation and structure analysis methods of natural polysaccharides of plants and fungi: A review of recent development. Molecules.

[165] Gopinath V., Saravanan S., Al-Maleki A.R., Ramesh M., Vadivelu J. (2018). A review of natural polysaccharides for drug delivery applications: Special focus on cellulose, starch and glycogen. Biomed. Pharmacother..

[166] Fan S., Zhang J., Nie W., Zhou W., Jin L., Chen X., Lu J. (2017). Antitumor effects of polysaccharide from Sargassum fusiforme against human hepatocellular carcinoma HepG2 cells. Food Chem. Toxicol..

[167] Faccin-Galhardi L.C., Aimi Yamamoto K., Ray S., Ray B., Carvalho Linhares R.E., Nozawa C. (2012). The in vitro antiviral property of Azadirachta indica polysaccharides for poliovirus. J. Ethnopharmacol..

[168] Olafsdottir E.S., Ingólfsdottir K. (2001). Polysaccharides from lichens: Structural characteristics and biological activity. Planta Med..

[169] Yu Y., Shen M., Song Q., Xie J. (2018). Biological activities and pharmaceutical applications of polysaccharide from natural resources: A review. Carbohydr. Polym..

[170] Cumpstey I. (2011). Chemical modification of polysaccharides. Int. Sch. Res. Not..

[171] Auzély-Velty R. (2011). Self-assembling polysaccharide systems based on cyclodextrin complexation: Synthesis, properties and potential applications in the biomaterials field. Comptes Rendus Chim..

[172] Pushpamalar J., Veeramachineni A.K., Owh C., Loh X.J. (2016). Biodegradable polysaccharides for controlled drug delivery. ChemplusChem.

[173] Ramírez H.L., Valdivia A., Cao R., Torres-Labandeira J.J., Fragoso A., Villalonga R. (2006). Cyclodextrin-grafted polysaccharides as supramolecular carrier systems for naproxen. Bioorganic Med. Chem. Lett..

[174] Izawa H., Kawakami K., Sumita M., Tateyama Y., Hill J.P., Ariga K. (2013). β-Cyclodextrin-crosslinked alginate gel for patient-controlled drug delivery systems: Regulation of host-guest interactions with mechanical stimuli. J. Mater. Chem. B.

[175] Omtvedt L.A., Dalheim M., Nielsen T.T., Larsen K.L., Strand B.L., Aachmann F.L. (2019). Efficient grafting of cyclodextrin to alginate and performance of the hydrogel for release of model drug. Sci. Rep..

[176] Peng K., Cui C., Tomatsu I., Porta F., Meijer A.H., Spaink H.P., Kros A. (2010). Cyclodextrin/dextran based drug carriers for a controlled release of hydrophobic drugs in zebrafish embryos. Soft Matter.

[177] Li C., Luo G.F., Wang H.Y., Zhang J., Gong Y.H., Cheng S.X., Zhuo R.X., Zhang X.Z. (2011). Host-guest assembly of pH-responsive degradable microcapsules with controlled drug release behavior. J. Phys. Chem. C.

[178] Xiao W., Chen W.H., Zhang J., Li C., Zhuo R.X., Zhang X.Z. (2011). Design of a photoswitchable hollow microcapsular drug delivery system by using a supramolecular drug-loading approach. J. Phys. Chem. B.

[179] Hou X., Zhang W., He M., Lu Y., Lou K., Gao F. (2017). Preparation and characterization of β-cyclodextrin grafted N-maleoyl chitosan nanoparticles for drug delivery. Asian J. Pharm. Sci..

[180] Prabaharan M., Gong S. (2008). Novel thiolated carboxymethyl chitosan-g-β-cyclodextrin as mucoadhesive hydrophobic drug delivery carriers. Carbohydr. Polym..

[181] Zhang X., Wu Z., Gao X., Shu S., Zhang H., Wang Z., Li C. (2009). Chitosan bearing pendant cyclodextrin as a carrier for controlled protein release. Carbohydr. Polym..

[182] Zhao S., Lee J., Xu W. (2009). Supramolecular hydrogels formed from biodegradable ternary COS-g-PCL-b-MPEG copolymer with α-cyclodextrin and their drug release. Carbohydr. Res..

[183] Singh M.N., Hemant K.S.Y., Ram M., Shivakumar H.G. (2010). Microencapsulation: A promising technique for controlled drug delivery. Res. Pharm. Sci..

[184] Dong Z., Kang Y., Yuan Q., Luo M., Gu Z. (2018). H_2_O_2_-responsive nanoparticle based on the supramolecular self-assemble of cyclodextrin. Front. Pharmacol..

[185] Carrazana J., Jover A., Meijide F., Soto V.H., Tato J.V. (2005). Complexation of adamantyl compounds by β-cyclodextrin and monoaminoderivatives. J. Phys. Chem. B.

[186] Wintgens V., Nielsen T.T., Larsen K.L., Amiel C. (2011). Size-controlled nanoassemblies based on cyclodextrin-modified dextrans. Macromol. Biosci..

[187] Elgadir M.A., Uddin M.S., Ferdosh S., Adam A., Chowdhury A.J.K., Sarker M.Z.I. (2015). Impact of chitosan composites and chitosan nanoparticle composites on various drug delivery systems: A review. J. Food Drug Anal..

[188] Zhao F., Repo E., Yin D., Chen L., Kalliola S., Tang J., Iakovleva E., Tam K.C., Sillanpää M. (2017). One-pot synthesis of trifunctional chitosan-EDTA-β-cyclodextrin polymer for simultaneous removal of metals and organic micropollutants. Sci. Rep..

[189] Chen Y., Ye Y., Wang L., Guo Y., Tan H. (2012). Synthesis of chitosan C6-substituted cyclodextrin derivatives with tosyl-chitin as the intermediate precursor. J. Appl. Polym. Sci..

[190] Liu Y., Yu Z.L., Zhang Y.M., Guo D.S., Liu Y.P. (2008). Supramolecular architectures of β-cyclodextrin-modified chitosan and pyrene derivatives mediated by carbon nanotubes and their DNA condensation. J. Am. Chem. Soc..

[191] Hu Y., Wu X.Y., JinRui X. (2018). Self-assembled supramolecular hydrogels formed by biodegradable PLA/CS diblock copolymers and β-cyclodextrin for controlled dual drug delivery. Int. J. Biol. Macromol..

